# Spatio-temporal habitat use by bobcats and their prey in the northern Sierra Madre Occidental, Chihuahua, Mexico

**DOI:** 10.1093/jmammal/gyag068

**Published:** 2026-08-24

**Authors:** Natalia Martínez-Sánchez, Alejandro González-Bernal, Zaira Y González-Saucedo, Hibraim A Pérez-Mendoza, Rurik List, Enrique Martínez-Meyer

**Affiliations:** Posgrado en Ciencias Biológicas, Universidad Nacional Autónoma de México, Ciudad Universitaria, Ciudad de México 04510, Mexico; Departamento de Zoología, Instituto de Biología, Universidad Nacional Autónoma de México, Ciudad Universitaria, Ciudad de México 04510, México; Departamento de Zoología, Instituto de Biología, Universidad Nacional Autónoma de México, Ciudad Universitaria, Ciudad de México 04510, México; World Wildlife Fund México, Av. Insurgentes Sur 1216, Despacho 702-703-704, Colonia Del Valle, Alcaldía Benito Juárez C.P., Ciudad de México 03100, México; Departamento de Zoología, Instituto de Biología, Universidad Nacional Autónoma de México, Ciudad Universitaria, Ciudad de México 04510, México; Laboratorio de Ecología Evolutiva y Conservación de Anfibios y Reptiles, Facultad de Estudios Superiores Iztacala, Universidad Nacional Autónoma de México, Tlalnepantla de Baz, Estado de México 54090, México; Área Académica en Biología de la Conservación, Departamento de Ciencias Ambientales, Universidad Autónoma Metropolitana Lerma, Av. De las Garzas 10, El Panteón, Lerma, Estado de México 52005, México; Departamento de Zoología, Instituto de Biología, Universidad Nacional Autónoma de México, Ciudad Universitaria, Ciudad de México 04510, México

**Keywords:** daily activity, lagomorph, predator–prey, spatial modeling, space use, temperate forest, actividad diaria, bosque templado, depredador-presa, lagomorfos, modelación espacial, uso del espacio

## Abstract

Bobcats (*Lynx rufus*) can adjust their hunting strategies in response to prey availability and environmental conditions. Understanding these dynamics over time provides valuable insights into predator–prey relationships and helps guide effective habitat management and conservation strategies. We investigated the spatial–temporal patterns of bobcats and 3 prey groups (lagomorphs, tree squirrels, and ground squirrels) in northern Chihuahua from November 2017 to January 2019, using camera traps and occupancy models. We examined the effect of habitat features, Coyote (*Canis latrans*), and cattle occurrence on bobcat and prey space use including their mutual effects across 3 seasons: cold-dry, warm-dry, and rainy. Additionally, we calculated the overlap in daily activity patterns between bobcats and their prey using kernel density estimates. The probability of bobcats using space was positively correlated with lagomorph space-use patterns during the cold-dry season of 2017 and the rainy season of 2018. Lagomorphs were mainly found on flat terrain for most of the year and away from water during the rainy season of 2018, while tree squirrels were near trails in the cold-dry season of 2017. Ground squirrels were found near water, with higher detection at sites of greater Bobcat activity during the rainy season of 2018. We did not find evidence that coyotes or cattle negatively affected space use or detection of bobcats and their prey. Bobcats had the highest temporal overlap with lagomorphs during the warm-dry and rainy seasons of 2018. Our results highlight the key role of lagomorphs in shaping the spatio-temporal patterns of bobcats. Habitat management should maintain suitable cover in flat terrain and riparian areas that support prey species to ensure Bobcat persistence.

## Teaser

This study reveals how prey shape the spatiotemporal patterns of bobcats in the northern Sierra Madre Occidental. We found that bobcats were closely associated with lagomorphs year-round, with the strongest alignment in space and time during the rainy season.

## Resumen

Los linces rojos (*Lynx rufus*) pueden ajustar sus estrategias de caza en respuesta a la disponibilidad de presas y a las condiciones ambientales. Comprender estas dinámicas a lo largo del tiempo proporciona valiosas perspectivas sobre las relaciones depredador-presa y ayuda a guiar las estrategias efectivas de manejo y conservación del hábitat. En este estudio investigamos los patrones espaciales y temporales de los linces rojos y tres grupos de presas (lagomorfos, ardillas arborícolas y ardillas terrestres) en el norte de Chihuahua, desde noviembre de 2017 hasta enero de 2019, utilizando cámaras trampa y modelos de ocupación. Examinamos los efectos de las características del hábitat y la presencia de coyotes (*Canis latrans*) y ganado sobre el uso del espacio de los linces rojos y sus presas, incluyendo sus efectos recíprocos a lo largo de tres temporadas: fría-seca, cálida-seca y lluviosa. Además, calculamos el solapamiento en los patrones de actividad diaria entre los linces y sus presas utilizando estimaciones de densidad de kernel. La probabilidad de que los linces rojos usaran un área se correlacionó positivamente con el uso del espacio por parte de los lagomorfos durante la temporada fría-seca de 2017 y la temporada de lluvias de 2018. Los lagomorfos se encontraron principalmente en terrenos planos durante la mayor parte del año y alejados de fuentes de agua durante la temporada de lluvias de 2018, mientras que las ardillas arborícolas se localizaron cerca de los senderos en la temporada fría-seca de 2017. Las ardillas terrestres se encontraron cerca del agua, con una mayor detección en sitios con mayor actividad de linces rojos durante la temporada de lluvias de 2018. No encontramos evidencia de que los coyotes o el ganado afecten negativamente el uso del espacio o la detección de los linces rojos y sus presas. Los linces rojos tuvieron el mayor solapamiento temporal con los lagomorfos durante las temporadas cálida-seca y de lluvias de 2018. Nuestros resultados destacan el papel clave de los lagomorfos en la configuración de los patrones espacio-temporales de los linces rojos. La gestión del territorio debería mantener una cobertura adecuada en zonas planas y áreas ribereñas que sustentan a las especies presa para garantizar la persistencia del lince rojo.

Predator–prey interactions play a key role in regulating the populations of species within both trophic guilds, directly influencing their distribution, behavior, and abundance (Sih 1984; Lima and Dill 1990; Lima 1998). For a predator to find prey, both must be present in the same space and time (Suraci et al. 2022). The ability of predators to detect and capture prey, and of prey to avoid predators, depends on a combination of behavioral and physical traits, as well as on resource availability (Schmitz 2017). This relationship is dynamic, with predators and prey responding to one another (Sih 1984; Lima and Dill 1990). Prey use areas that provide optimal food sources, refuges, and escape routes allow prey to meet their requirements while avoiding periods of high predator activity to minimize risk (Lima and Dill 1990). On the other hand, predators concentrate their efforts in areas and times where prey are most available to maximize hunting success (Sih 1984; Halle 2000; Monterroso et al. 2013).

Habitat structure such as vegetation cover and terrain configuration influences the visibility and mobility of both predator and prey (Lima and Dill 1990; Gorini et al. 2012). For example, in areas with dense vegetation and irregular terrain, prey find more hiding spots, which can reduce predation risk (Lima and Dill 1990; Gorini et al. 2012; Smith et al. 2019). These conditions favor ambush predators, who remain concealed while approaching. In open areas, prey are more visible but can detect predators earlier and flee quickly; consequently, some predators employ active pursuit (Gorini et al. 2012; Smith et al. 2019).

Changes in temperature and precipitation across seasons modify the availability of key resources such as water, food, and cover, triggering cascading effects on prey species and the predators that rely on them (Bender et al. 2017; Svoboda et al. 2019; Vettorazzi et al. 2022). During harsh weather conditions, reduced plant productivity restricts resources to a few accessible sites, causing herbivores to aggregate and become more predictable to predators (Hernández et al. 2011; Basille et al. 2013). Many predators such as bobcats (*Lynx rufus*), can switch prey (within their physiological restrictions, e.g., strict carnivory) according to local conditions, focusing their hunting efforts on the most abundant or vulnerable species at particular sites and times (Lariviere and Walton 1997; Bastille-Rousseau et al. 2016). Bobcats primarily feed on lagomorphs (hares and rabbits) and rodents; other prey, including birds, reptiles, and even larger mammals such as White-tailed Deer (*Odocoileus virginianus*) are occasionally consumed (Lariviere and Walton 1997; Aranda et al. 2002; López-Vidal et al. 2014). This dietary flexibility allows them to persist across diverse environmental conditions, from temperate forests to semi-arid areas throughout North America (Lariviere and Walton 1997).

Bobcats are common predators in the Sierra Madre Occidental (SMO), a mountain range spanning western Mexico that supports the highest density of temperate forests in the country (López-González and García-Mendoza 2012; CONABIO 2014). The region is characterized by rugged terrain, steep elevational gradients, and marked seasonality, which together create a highly heterogeneous ecological landscape (CONABIO 2014). Nevertheless, it faces pressures from human activities, particularly extensive cattle grazing, which can alter habitat structure and affect prey availability and distribution (CONABIO 2014; Caldwell and Klip 2023). Coyotes (*Canis latrans*) are another abundant predator in the SMO and may act as potential competitors to bobcats, as both species commonly exploit similar prey (Fedriani et al. 2000; Melville et al. 2020). This dietary overlap could lead to resource competition, particularly when prey availability is low (Dyck et al. 2022). Understanding how bobcats use their habitat and respond to seasonal variation in prey availability is essential for developing adaptive management and conservation strategies, especially considering how human activities may disrupt these ecological dynamics.

Although bobcats have been widely studied, little is known about their ecology in the temperate forests of the northern SMO. In Mexico, most studies on bobcats have been conducted in arid regions and have focused on dietary analysis using scats (Delibes and Hiraldo 1987; Delibes et al. 1997; López-Vidal et al. 2014), while few have integrated spatial or temporal analyses of prey availability (Serna-Lagunes et al. 2019; Andrade-Ponce et al. 2022). In this context, camera traps provide direct and non-intrusive observations of predators and their prey over time and across multiple locations (Keim et al. 2019; Smith et al. 2020). These data can be analyzed via occupancy modeling to determine how prey availability influences the spatio-temporal patterns of predators (Mackenzie et al. 2017).

In this study, we combined camera traps and occupancy models to examine the spatial and temporal relationships between bobcats and their prey in the northern SMO. First, we assessed seasonal patterns of space use by bobcats and their potential prey—lagomorphs, tree squirrels, and ground squirrels—in relation to habitat features and the occurrence of coyotes and cattle. To explore predator–prey interactions, we included prey and bobcat occurrences as covariates. Second, we compared the daily activity patterns between pairs of species.

Because lagomorphs are primary prey for bobcats throughout their range, we hypothesized stronger associations between bobcat space use/activity and lagomorphs than with other prey. We further hypothesized that cattle activity would reduce lagomorph space use and activity via trampling and reduced forage availability, thereby indirectly reducing bobcat space use and activity.

## Methods

### Study area

We conducted this study on 2 private properties and a section of a communal property, namely ejido El Largo, located between the municipalities of Casas Grandes and Madera in northern Chihuahua, Mexico (Fig. 1; 30°2′N, 108°27′W). Our study area encompassed approximately 150 km^2^, characterized by the mountains of the SMO with elevations ranging from 1,689 masl to 2,461 masl. The climate is temperate sub-humid (García 1998), with temperatures ranging from −5.1 °C in January to 31.1 °C in July (Cuervo-Robayo et al. 2020). Annual precipitation averages 773.6 mm (Cuervo-Robayo et al. 2020), with most of the rainfall occurring between July and October and light snowfall from December to February. Land cover consists primarily of coniferous forest (72%), followed by deciduous forest (25%). Other land cover types included cropland (2%) and grassland (1%; CONABIO 2020). The primary land use is cattle grazing.

**Fig. 1:**
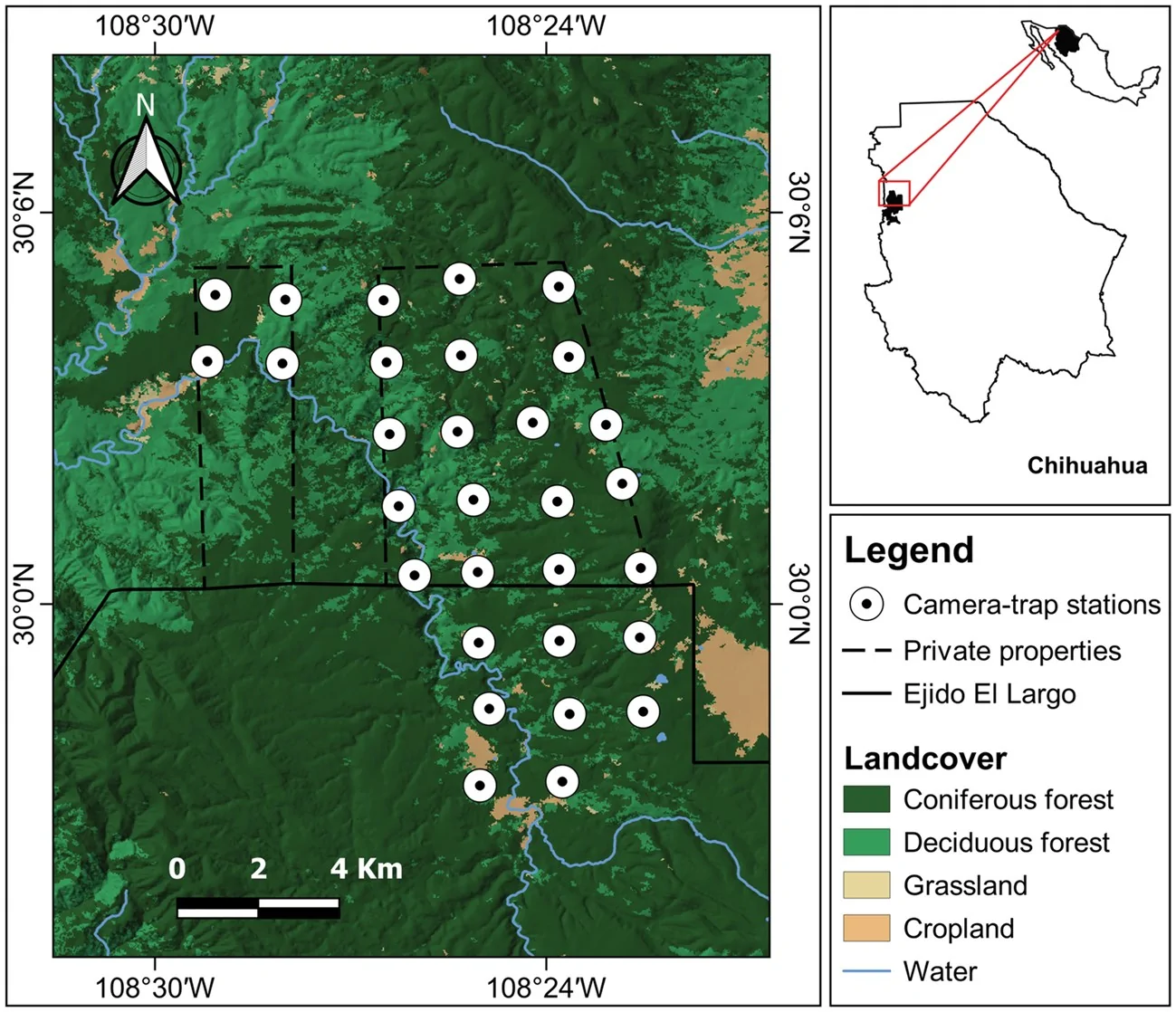
Location of the study area in northern Sierra Madre Occidental, Chihuahua, Mexico, 2017 to 2019.

Potential prey species present included 2 tree squirrels (Order Rodentia; Subfamily Sciurinae), Abert’s Squirrel (*Sciurus aberti*) and Mexican Fox Squirrel (*S. nayaritensis*); 2 ground squirrels (Order Rodentia; Subfamily Xerinae), Rock Squirrel (*Otospermophilus variegatus*) and Cliff Chipmunk (*Neotamias dorsalis*); 2 lagomorphs (Order Lagomorpha), Black-tailed Jackrabbit (*Lepus californicus*) and Eastern Cottontail (*Sylvilagus floridanus*); White-tailed Deer (*Odocoileus virginianus*), and some small rodents. The medium and large carnivore community is comprised of Bobcat, Coyote, Gray Fox (*Urocyon cinereoargenteus*), Puma (*Puma concolor*), Black Bear (*Ursus americanus*), and occasionally Mexican Wolf (*C. lupus baileyi*).

### Data collection

We conducted a camera trap survey from November 2017 to January 2019. We deployed 30 Browning BTC-6HD Elite camera traps in a grid arrangement, spaced 2 km apart to ensure spatial independence. We attached cameras to trees at a height of 0.5 m above the ground and equipped them with 32 GB memory cards and lithium batteries for long-term operation. We configured them to take 3 consecutive photos with a 1 min delay between triggers and checked them every 7 to 8 mo to retrieve memory cards and replace batteries. Cameras remained active 24 h per day throughout the entire study period.

We organized and tagged the photographs using the photo manager DigiKam (version 7.2.0; Digikam Team 2021). For this study, we focused on the most common prey species, categorizing them into 3 groups: lagomorphs, tree squirrels, and ground squirrels. We excluded other small rodents (e.g., mice, rats, and voles) due to the difficulty of detecting and identifying them from camera traps.

We defined 3 climatic seasons based on mean monthly precipitation and temperature: cold-dry (November to February); warm-dry (March to June); and rainy (July to October). We divided each season into 70 d sampling periods to capture environmental variation and maximize detections, assuming that habitat conditions remained constant within each season. For occupancy modeling, we considered consecutive photographs of the same species taken within a 24-h period as a single detection to avoid pseudoreplication (Jones et al. 2016; Cepeda-Duque et al. 2023). To create species-specific binary detection histories (1 = detected, 0 = not detected), we collapsed detections into 7 d sampling occasions with 10 replicates using the R package “camtrapR” (Niedballa et al. 2016).

### Habitat covariates

We used the packages “raster” (Hijmans 2021) and “landscapemetrics” (Hesselbarth et al. 2019) in R (R Development Core Team 2021) to characterize each camera site with 9 habitat covariates (Supplementary Data SD1). We measured the Euclidean distance from each camera site to the nearest trail and water source (e.g., streams, rivers, and ponds) using 1:50,000 topographic maps of Chihuahua (INEGI 2018). To represent habitat preferences, we defined a 500 m buffer for bobcats and a 100 m buffer for prey around each camera site. Within these buffers, we calculated mean elevation, slope, vector ruggedness measurement (VRM; Sappington et al. 2007), and seasonal normalized difference vegetation index (NDVI), as well as the percentage of canopy cover and the percentages of the 2 most predominant land cover types (coniferous and deciduous forests). We derived elevation, VRM, and slope from the digital elevation model of Mexico (INEGI 2013). We extracted NDVI values from the Landsat 8 Collection 1 Tier 1 8-d NDVI Composite in Google Earth Engine (Gorelick et al. 2017). We calculated canopy cover as the percentage of the buffer area with tree canopy cover greater than 10% using the Global 2010 Tree Cover layer from the Global Forest Change dataset (Hansen et al. 2013) and obtained land cover percentages from the 2015 Mexico land cover layer (CONABIO 2020). The highest resolution available for most layers was 30 m; for consistency, we resampled all other layers to this resolution and used them as base maps.

### Occupancy modeling

We used single-species, single-season occupancy models to assess seasonal patterns of space use by bobcats and their prey in each season. These models estimate the probability that a site is occupied or used by a species (*ψ*) and the probability of detecting the species at a site during each sampling occasion given its presence (*P*), while accounting for imperfect detection (MacKenzie et al. 2017). Due to our limited sample size, we did not use multi-season or multi-species models to avoid overparameterization and potential convergence issues (Jones et al. 2016; Wait et al. 2018). Instead, we estimated the conditional occupancy probability of bobcats, their prey species (lagomorphs, tree squirrels, and ground squirrels), coyotes, and cattle at each camera site during each season, and used these estimates as species occurrence covariates to examine interspecific interactions (Jones et al. 2016; Massara et al. 2018; Wait et al. 2018). Conditional occupancy (Ψ_cond_) is the probability that the species was present at a site during a season, given it was not detected; if a species was detected at least once, Ψ_cond_ = 1 (Mackenzie et al. 2017). We also calculated detection rates for cattle, coyotes, and bobcats as an index of activity by dividing the number of independent detections at each camera site by the number of camera nights during each season (Caldwell and Klip 2023).

To identify which factors influenced the probability of occupancy (*ψ*) and detection (*P*) of bobcats and their prey in each season, we used a stepwise approach based on the biology of each species (Supplementary Data SD1). First, we assessed species-specific effects of individual habitat covariates and the detection rates of cattle, coyotes, and bobcats (only in prey models) on the probability of detection, including a null model (intercept-only), while holding occupancy constant. We retained the most supported detection model to estimate the probability of occupancy in 2 stages, which reduced model complexity.

In the first stage, we fit univariate models to identify the most important habitat feature for each species by assessing species-specific effects of individual habitat features on the probability of occupancy, including a null model (intercept-only). We then used the top-ranked model to estimate the probability conditional occupancy of bobcats and prey. We followed the same procedure for coyotes and cattle. In the second stage, we retained the best habitat variable and fitted multi-variable models using only biologically plausible combinations of up to 3 additional covariates, which varied by species and season to reflect their ecological and seasonal relevance. We combined habitat and species-occurrence covariates to evaluate the seasonal drivers of space use and interspecific interactions.

For bobcats, we specifically included each prey occurrence (lagomorphs, tree squirrels, and ground squirrels) to assess prey availability, coyote occurrence to evaluate competition, and cattle occurrence to assess potential disturbance effects. For species occupancy of prey, we included Bobcat and Coyote occurrence to evaluate predation risk, as well as cattle occurrence to assess competition. However, we only included cattle in the occupancy models for lagomorphs and ground squirrels, as these species share similar feeding habits. We excluded ground squirrels from the cold-dry season because both species in the study area hibernate (Oaks et al. 1987; Hart 1992; Kilanowski and Koprowski 2016).

Before modeling, we z-standardized all covariates (mean = 0, SD = 1). We then assessed collinearity among covariates using Pearson’s correlation coefficient (|*r*| > 0.70). We found strong correlations between slope and VRM within 500 m (*r *= 0.90), and between elevation and NDVI within 500 m during the cold-dry season of 2018 (*r *= 0.91). To avoid multicollinearity, we did not include these highly correlated variable pairs together in the same multi-variable models for bobcats.

We implemented occupancy models in a Bayesian framework using the R package “ubms” (Kellner et al. 2022). For each model, we ran 3 parallel Markov Chain Monte Carlo chains of 10,000 iterations, discarding the first 5,000 iterations as burn-in. We used uninformative default Logistic (0, 1) priors for intercepts and regression coefficients. We checked model convergence through the Gelman–Rubin statistic (Rhat < 1.1; Gelman and Rubin 1992). We compared models using leave-one-out cross-validation and ranked them in descending order by their expected log-pointwise predictive density (ELPD), which measures predictive accuracy (Vehtari et al. 2017). We considered a model to have less support if its absolute difference in ELPD from the top model was larger than the standard error of that difference (Kellner et al. 2022). We assessed the goodness of fit of the most supported multi-variable models using the MacKenzie-Bailey χ^2^ test (MacKenzie and Bailey 2004), where a Bayesian *P* value close to 0 or 1 indicates a poor fit (Gelman et al. 2014; MacKenzie et al. 2017). We considered a covariate to have strong support if its 95% credible interval (CI) excluded zero. When the same covariate had strong support in multiple models, we reported its coefficient (β) and corresponding 95% CI from the model with the highest weight. We interpreted occupancy as “use,” given that bobcats can move larger distances daily (4.9 km; Elizalde-Arellano et al. 2012) than the spacing between camera sites. Therefore, the same individual could be detected at multiple sites within a sampling period, which may not reflect true occupancy (Kéry and Royle 2016; MacKenzie et al. 2017).

### Temporal analysis

We employed kernel density estimation (Ridout and Linkie 2009) to quantify the daily activity patterns of bobcats and their prey across seasons and calculated their similarity using the coefficient of overlap (Δ) in the R package “Overlap” (Meredith and Ridout 2020). To avoid overestimation of activity periods when multiple pictures of the same individual were taken in short periods of time, we considered consecutive photographs of the same species taken at least 60 min apart (Lewis et al. 2021). We selected the Δ_1_ estimator for small sample sizes (<50), with values ranging from 0 (no overlap) to 1 (total overlap; Ridout and Linkie 2009). We calculated 95% confidence intervals (CI) for the overlap coefficients with 10,000 bootstrap samples. We assessed the differences in daily activity patterns between species pairs using the nonparametric Watson’s 2-sample test of homogeneity (*U*^2^) in the R package “Circular” (Agostinelli and Lund 2017).

## Results

We obtained 1,261 independent detections of our 6 target taxa across 7,508 trap nights over 4 seasons, including cattle (*n *= 469), lagomorphs (*n *= 243), tree squirrels (*n *= 231), coyotes (*n *= 143), bobcats (*n *= 92), and ground squirrels (*n *= 83). The proportion of cameras where bobcats and their prey were detected at least once varied across seasons: 0.30 to 0.43 for bobcats; 0.43 to 0.50 for lagomorphs; and 0.43 to 0.60 for tree squirrels. For ground squirrels, the proportion remained between 0.40 and 0.43 (Table 1).

**Table 1:** Proportion of cameras where the species were detected across seasons in northern Sierra Madre Occidental, Chihuahua, Mexico, 2017 to 2019.

Species	Cold-dry 2017	Warm-dry 2018	Rainy 2018	Cold-dry 2018
**Tree squirrel**	0.60	0.43	0.47	0.53
**Ground squirrel^a^**		0.40	0.43	
**Lagomorph**	0.50	0.43	0.33	0.43
**Bobcat**	0.37	0.43	0.30	0.40
**Coyote**	0.63	0.30	0.50	0.43
**Cattle**	0.50	0.60	0.60	0.67

a Ground squirrels were not detected in cold-dry seasons because they hibernate from November to February (Oaks et al. 1987; Hart 1992).

Top-ranked models for each species and season exhibited an acceptable fit, with *P* value ranging from 0.12 to 0.81 (Supplementary Data SD2). Mean estimates of the probability of use and detection from the top-ranking multi-variable models varied across species and seasons (Fig. 2; Supplementary Data SD3). Bobcats showed the highest probability of use during the warm-dry (*ψ*  =  0.74, CI = 0.40 to 0.98) and cold-dry seasons (*ψ*  =  0.75, CI = 0.47 to 0.97) of 2018, and the lowest probability of use during the rainy season of the same year (*ψ*  =  0.49, CI = 0.18 to 0.87), while detection remained constant across seasons. For lagomorphs, the highest probability of use was in the cold-dry season of 2018 (*ψ*  =  0.61, CI = 0.35 to 0.85) and the lowest in the cold-dry season of 2017 (*ψ*  =  0.34, CI = 0.11 to 0.61), with the highest detection during the cold-dry season of 2017 (*P *= 0.52, CI = 0.43 to 0.61), and the lowest in the rainy season of 2018 (*P *= 0.12, CI = 0.05 to 0.24). Tree squirrels had the highest probability of use during the cold-dry seasons of 2017 and 2018 (*ψ*  =  0.71, CI = 0.50 to 0.90 and *ψ*  =  0.74, CI = 0.52 to 0.91, respectively), the lowest in the warm-dry season of 2018 (*ψ*  =  0.45, CI = 0.24 to 0.67), and a slightly higher probability of detection in the cold-dry season of 2018 (*P *= 0.36, CI = 0.27 to 0.44). The probability of space use by ground squirrels remained constant, with the highest detection probability during the rainy season of 2018 (*P *= 0.24, CI = 0.16 to 0.34).

**Fig. 2:**
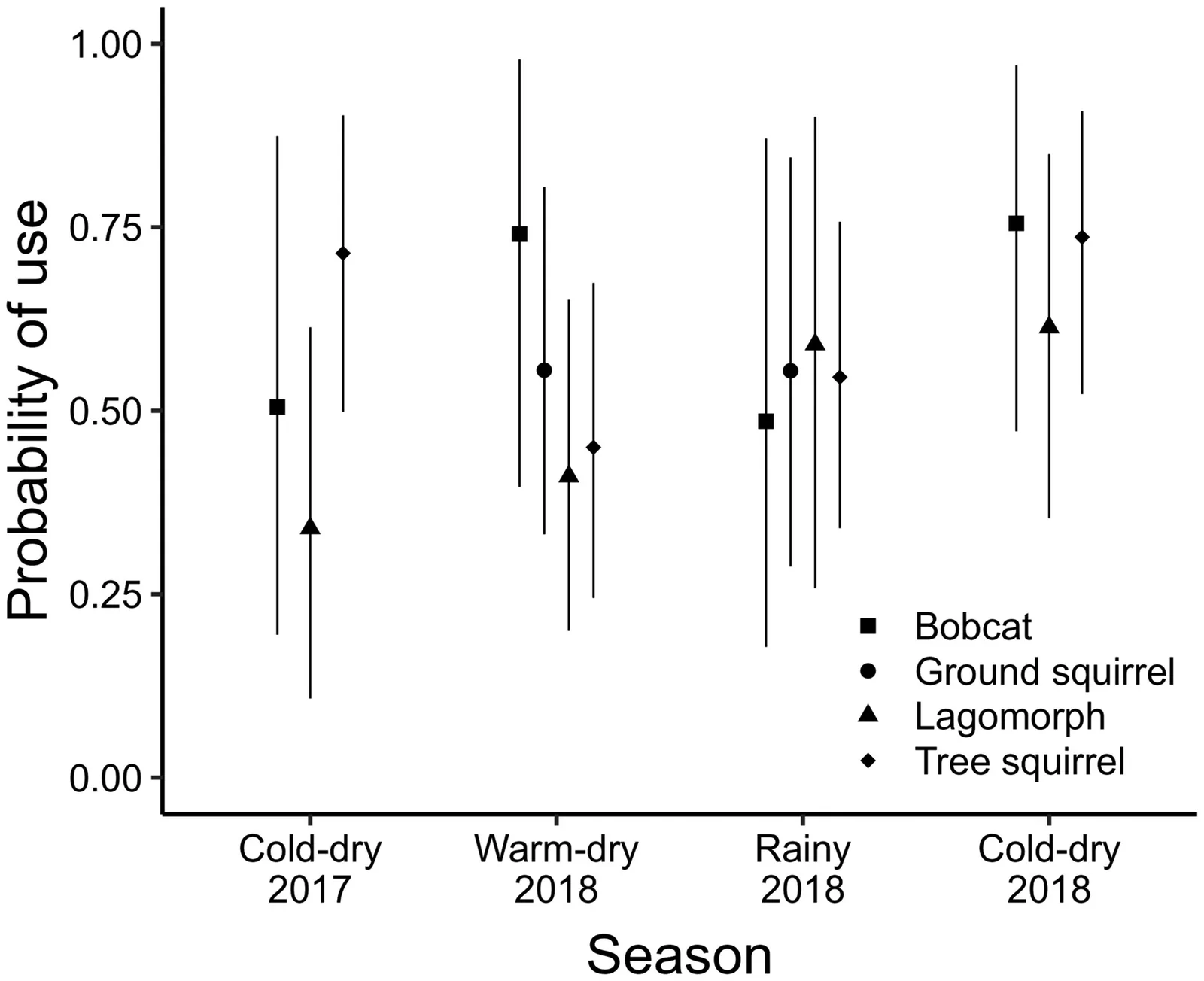
Mean estimates of the probability of use with 95% credible intervals for bobcats and their prey, derived from the top multi-variable models for each season in northern Sierra Madre Occidental, Chihuahua, Mexico (2017 to 2019).

### Bobcat

During the cold-dry seasons of 2017 and 2018, cattle detection rate had a strong positive effect on bobcat detection (β  =  0.65, CI = 0.08 to 1.20; and β  =  0.40, CI = 0.03 to 0.77, respectively; Supplementary Data SD3). In the warm-dry and rainy seasons of 2018, the null models were the best-ranked, indicating that no covariates influenced detection. Habitat covariates in the best univariate models varied among seasons: deciduous forest cover (β = −1.87, CI = −4.32 to −0.31; Fig. 3A) had a negative effect on bobcat space use during the cold-dry season of 2017 with strong support; while canopy cover (β = −1.28, CI = −3.96 to 1.04) in the warm-dry season of 2018 and distance to water (β  =  1.11, CI = −0.54 to 2.90) in the cold-dry season of 2018 showed a weak negative effect; and no single habitat variable was informative in the rainy season of 2018. In multi-variable models, lagomorph occurrence was consistently included in the best-ranked models across all seasons (Supplementary Data SD2) with a strong positive effect on bobcat space use during the cold-dry season of 2017 (β  =  1.69, CI = 0.09 to 3.68; Fig. 3A) and the rainy season of 2018 (β  =  2.17, CI = 0.37 to 4.86; Fig. 3B). In contrast, tree squirrels (β = −1.68, CI = −3.66 to −0.14; Fig. 3A) had a strong negative effect in the cold-dry season of 2018, whereas ground squirrels were included in the top-ranked models during the warm-dry and rainy seasons of 2018 but did not show strong support (Supplementary Data SD2).

**Fig. 3:**
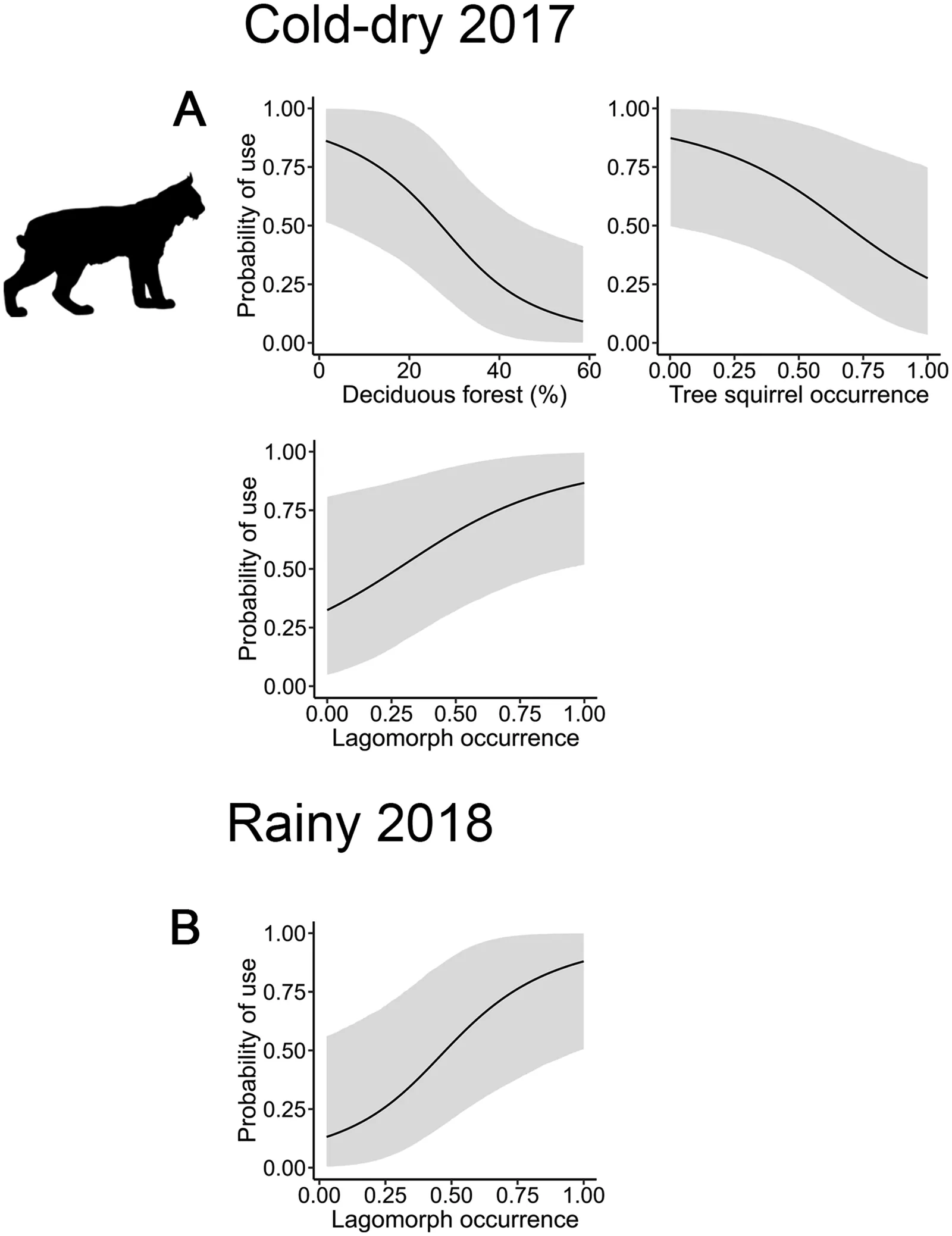
Covariate effects on the probability of use (*ψ*) by bobcats across seasons in northern Sierra Madre Occidental, Chihuahua, Mexico, 2017 to 2019. Only covariates with strong support (95% credible intervals not crossing zero) from the top univariate and multi-variable occupancy models with the highest weight for the cold-dry 2017 (A) and rainy 2018 (B) seasons are shown. Gray shaded area represents the 95% credible intervals.

### Lagomorphs

The best detection models for lagomorphs varied across seasons, with multiple factors affecting their detection (Supplementary Data SD3). Elevation had a strong positive effect during the cold-dry season of 2017 (β  =  0.48, CI: 0.19 to 0.80), coyote detection rate a weak negative effect during the warm-dry season of 2018 (β  =  0.37, CI = −0.15 to 0.87), VRM showed a strong negative effect in the rainy season of 2018 (β = −1.38, CI = −2.52 to −0.28), and bobcat detection rate (β  =  0.58, CI = 0.28 to 0.91) had a strong positive effect during the cold-dry season of 2018. In univariate models, VRM was included in the top-ranked models for the cold-dry season of 2017 (β = −3.19, CI = −5.72 to −1.33), for the warm-dry (β = −1.11, CI = −2.39 to −0.10) and the cold-dry seasons of 2018 (β = −2.42, CI = −4.52 to −0.85; Fig. 4), consistently showing a strong negative effect on space use, whereas distance to the nearest water source (β  =  1.39, CI = 0.13 to 3.04; Fig. 4D) had a strong positive effect in the rainy season of 2018. Across all seasons, bobcat occurrence was included in top-ranked multi-variable models, showing a strong positive effect on lagomorph space use during the cold-dry season of 2017 (β  =  1.50, CI = 0.19 to 3.12; Fig. 4A) and the rainy season of 2018 (β  =  2.03, CI = 0.37 to 4.50; Fig. 4D).

**Fig. 4:**
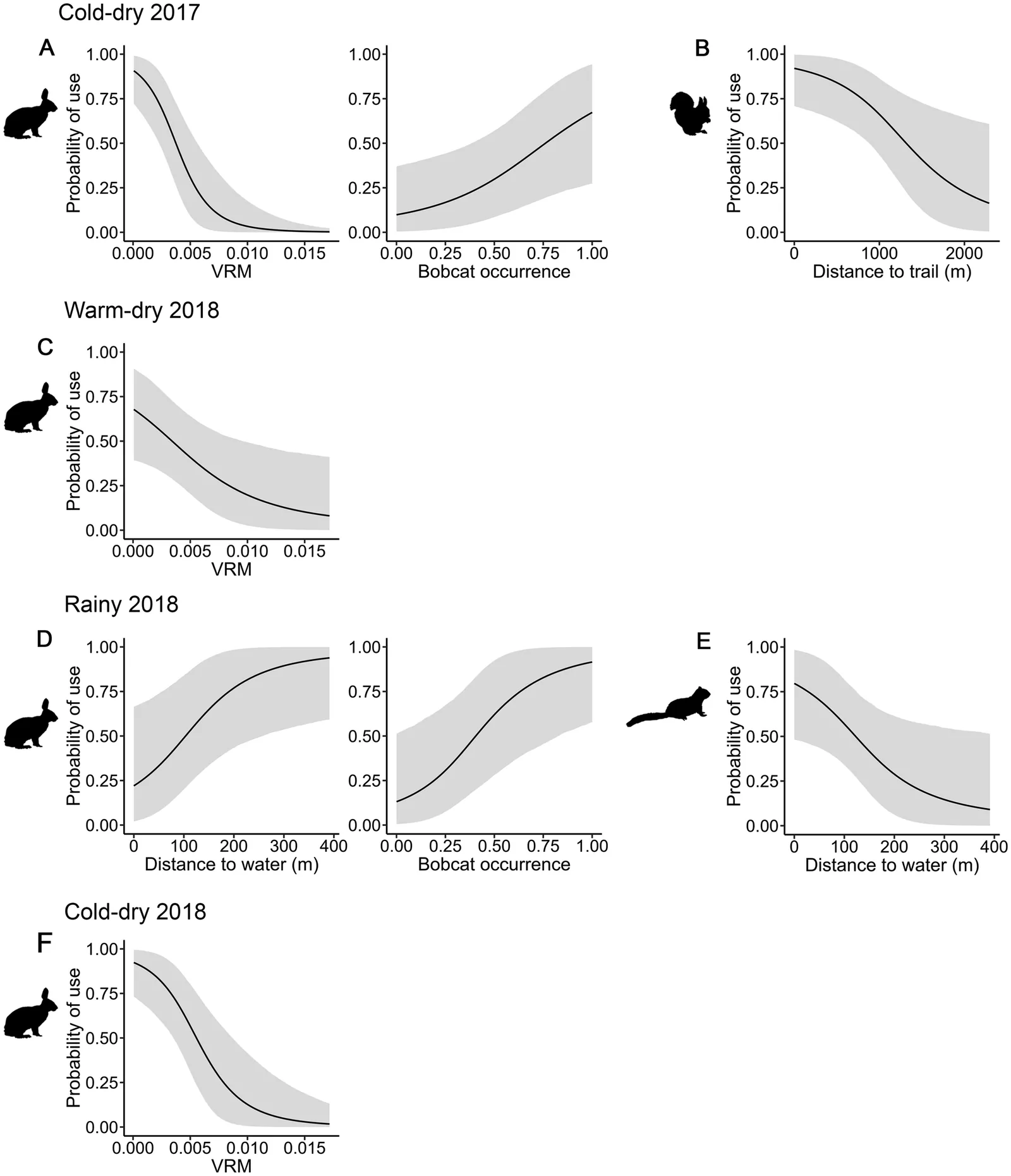
Covariate effects on the probability of use (*ψ*) by prey across seasons in northern Sierra Madre Occidental, Chihuahua, Mexico, 2017 to 2019. Prey groups included lagomorphs (A, C, D, and F), tree squirrels (B), and ground squirrels (E). Only covariates with strong support (95% credible intervals not crossing zero) from the top univariate and multi-variable occupancy models with the highest weight are shown. Gray-shaded area represents the 95% credible intervals.

### Tree squirrel

Detection of tree squirrels showed a strong positive effect of slope during the cold-dry season of 2017 (β  =  0.56, CI = 0.19 to 0.95; Supplementary Data SD3). In contrast, distance to the nearest water source had a negative effect, with strong support during the warm-dry season of 2018 (β = −0.83, CI = −1.51 to −0.26) and weak support during the rainy season of 2018 (β = −0.36, CI = −0.82 to 0.04), while elevation had a strong negative effect in the cold-dry season of 2018 (β = −0.82, CI = −1.24 to −0.43; Supplementary Data SD3). In univariate models, null models received the most support for the cold-dry season of 2017 and the warm-dry and rainy seasons of 2018, while canopy cover had a weak negative effect during the cold-dry season of 2018 (β = −0.62, CI = −1.60 to 0.28), suggesting that no single habitat covariate had a strong effect on tree squirrel space use. In the top-ranked multi-variable model for the cold-dry season of 2017, distance to the nearest trail had a strong negative effect on space use (β = −1.44, CI = −2.99 to −0.24; Fig. 4B), whereas bobcat occurrence had a negative effect but lacked support (β = −0.98, CI = −2.24 to 0.09). No additional covariates received support in the best-ranked multivariable models for the warm-dry, rainy, and cold-dry seasons of 2018.

### Ground squirrel

During the warm-dry season of 2018, distance to the nearest water source exhibited a weak negative effect on ground squirrel detection (β = −0.61, CI = −1.35 to 0.12). On the other hand, Bobcat detection rate had a strong positive effect during the rainy season of 2018 (β  =  0.67, CI = 0.35 to 1.00; Supplementary Data SD3). In both univariate and multivariable models for the warm-dry season of 2018, the null model was best ranked, indicating no effect of any covariate on ground squirrel space use. However, Bobcat occurrence was included in the subsequent competitive models without support (Supplementary Data SD2). In contrast, distance to the nearest water source (β =−1.22, CI = −2.80 to −0.07; Fig. 4E) was included in the top-ranked univariate model during the rainy season of 2018, showing a strong negative effect on ground squirrel space use. In the multi-variable models, no other covariate exhibited strong effects.

### Temporal overlap

Bobcats showed cathemeral behavior, being active both day and night, with the highest activity occurring around crepuscular hours during the cold-dry seasons of 2017 and 2018, as well as during the rainy season of 2018 (Fig. 5). In contrast, during the warm-dry season of 2018, bobcats displayed nocturnal activity. Lagomorphs were predominantly nocturnal across all seasons, mostly active at dawn and dusk, while both squirrel groups were exclusively diurnal, with peak activity from morning to midday (Fig. 5).

**Fig. 5:**
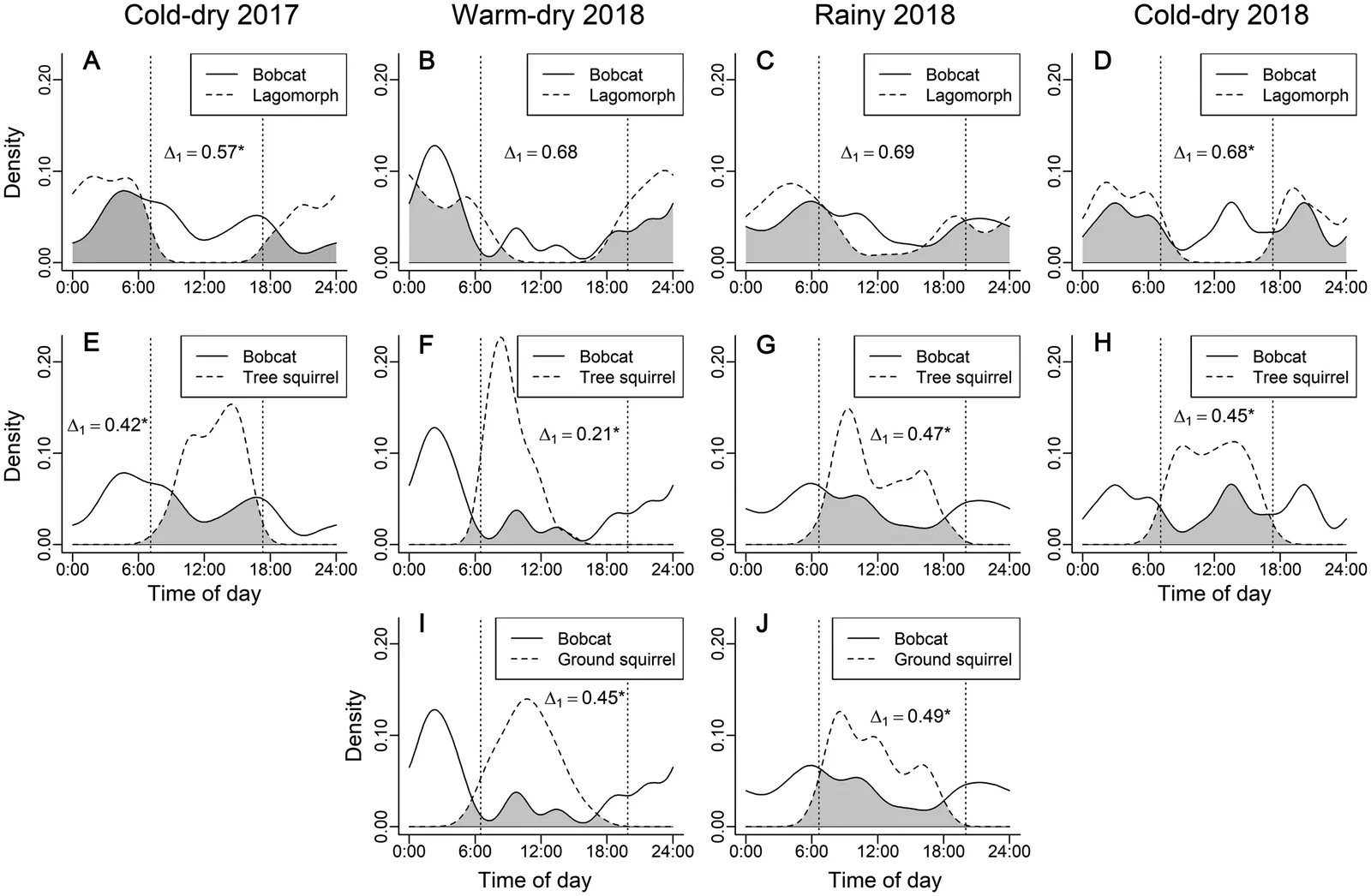
Overlap in activity patterns between bobcats (solid line) and their prey (dashed line) across seasons in northern Sierra Made Occidental, Chihuahua, Mexico, 2017 to 2019. Prey groups included lagomorphs (A–D), tree squirrels (E–H), and ground squirrels (I and J). Gray-shaded area represents overlap. Asterisk (*) indicates *P *< 0.05 from the Watson *U*^2^ test. Vertical dotted lines represent sunrise and sunset times for each season.

Bobcats had higher temporal overlap with lagomorphs during the warm-dry season of 2018 (Δ_1_ = 0.68, CI = 0.47 to 0.83; Fig. 5B) and the rainy season of 2018 (Δ_1_ = 0.69, CI = 0.58 to 0.94; Fig. 5C), with no statistical differences between these seasons (*U* ^2^ = 0.09, *P *> 0.05 and *U* ^2^ = 0.07, *P *> 0.05, respectively). In the cold-dry season of 2018, we observed a notable overlap (Δ_1_ = 0.68, CI = 0.56 to 0.83; Fig. 5D) with significant differences in activity patterns (*U* ^2^ = 0.22, *P *< 0.05). Bobcats had lower overlap with tree squirrels in the warm-dry season of 2018 (Δ_1_ = 0.21, CI = 0.04 to 0.35; Fig. 5F), with significant differences in activity distributions (*U* ^2^ = 0.83, *P *< 0.05). Additionally, during the rainy season of 2018, there was moderate temporal overlap with ground squirrels (Δ_1_ = 0.49, CI = 0.25 to 0.68; Fig. 5J), which differed significantly (*U* ^2^ = 0.36, *P *< 0.05).

## Discussion

Our findings reveal that in the SMO bobcats primarily use areas with higher lagomorph occurrence throughout the year, closely aligning with lagomorph space use. However, this pattern varied among seasons and was not observed during the warm-dry and cold-dry seasons of 2018. In the warm-dry season of 2018, lagomorphs exhibited stable space use, suggesting that they remained available and that bobcats may have continued to rely on them. Nevertheless, the lack of a strong spatial relationship with lagomorphs or other species coincided with greater space use by bobcats, which may be driven by behaviors such as territorial patrols, dispersal movements, or mate searching (Chamberlain et al. 2003; McNitt et al. 2020; Melville et al. 2020). In contrast, the absence of a clear relationship during the cold-dry season of 2018 should be interpreted with caution, as the failure of some cameras led to the underrepresentation of certain areas and may not reflect a true ecological pattern.

Lagomorphs could prioritize areas with suitable vegetation cover, even if bobcats are present, because these areas provide food resources and hiding spots, increasing their chances of survival (Chapman et al. 1980; Bond et al. 2001). During the cold-dry season of 2018, lagomorph detection probability was higher at elevations above 2,000 m (Supplementary Data SD3), where coniferous forests dominated. Conifers offer better cover from wind and cold temperatures, reduce snow accumulation, and create more favorable microclimates during colder months (Lovallo and Anderson 1996; Sunquist and Sunquist 2002). Consequently, bobcats likely favored this land cover for hunting, as indicated by their use of areas with less deciduous cover, which were dominated by coniferous forests. This preference for coniferous forests has been attributed to greater concealment cover and increased prey availability during winter in the northern latitudes (McCord 1974; Lovallo and Anderson 1996). Within these forests, light to moderate cattle grazing may help clear snow from the ground and remove dry forage, promoting the growth of more palatable vegetation that attracts lagomorphs (Flinders and Hansen 1975; Holechek et al. 2003). As a result, higher detection of bobcats in areas with high cattle activity was likely due to bobcats following lagomorphs into these areas.

Lagomorphs consistently used flat terrain, facilitating their high-speed movement and allowing them to employ their characteristic zigzag fleeing pattern to evade predators (Chapman et al. 1980; Glebskiy et al. 2018). Flat areas also retain more soil moisture, favoring the growth of grasses that provide both cover and forage (Wilcox et al. 1988; Bond et al. 2001). However, bobcats can effectively hunt lagomorphs in these areas by using the surrounding vegetation to stealthily approach and ambush them (Sunquist and Sunquist 2002). In contrast, tree squirrels usually select steeper slopes for nesting in larger and taller trees (Edelman and Koprowski 2005; Ramos-Lara and Cervantes 2007). Although bobcats were not in areas frequented by tree squirrels during the cold-dry season, the proximity of tree squirrels to trails increases their predation risk. Bobcats often travel along these pathways, which provide clear and accessible routes that enable them to traverse steeper slopes (Reichman and Aitchison 1981; Lariviere and Walton 1997). Higher detection of tree squirrels on steeper slopes during this season suggests that they spend more time foraging on the ground, making them easier for bobcats to detect and capture in areas unsuitable for lagomorphs.

Bobcat space use decreased during the rainy season, likely because females hunt closer to their den sites to rear kittens (Lovallo and Anderson 1996; Chamberlain et al. 2003; Melville et al. 2020). The lush vegetation that grows after rainfall provides concealment cover and supports higher prey abundance, enhancing reproductive success and kitten survival (Tewes and Hornocker 2007). Bobcats likely hunt lagomorphs in areas far from water sources, where they were found. Lagomorphs consume succulent forage that provides sufficient water content, reducing their need to actively search for water (Schwartz and Schwartz 2001; Harris et al. 2015). In contrast, opportunities to hunt ground squirrels increased near water sources, where the terrain was rugged and rocky outcrops were common. These areas provide well-drained soils and sloped terrain for ground squirrels to burrow, dead tree stumps and open branches for early predator detection, and easier access to food resources from riparian vegetation (Oaks et al. 1987; Ortega 1987; Hart 1992). Both dense vegetation and rocky terrain serve as den sites and stalking cover for bobcats, potentially increasing encounters with ground squirrels, which could explain the higher detection probability of these squirrels in sites with high bobcat activity during the rainy season. Aranda et al. (2002) observed an increase in rock squirrel consumption during a similar wet period in a shrubland area of northern Mexico. Rock squirrels provide significant supplementary biomass (∼700 g; Oaks et al. 1987), supporting the rearing of kittens and aiding in their hunting skills development.

We did not find evidence that bobcats directly compete with coyotes. Recent studies suggest that the strength and direction of such interactions are context-dependent and may not always result in negative effects on bobcats, particularly in heterogeneous habitats (Svoboda et al. 2019; Dyck et al. 2022). Both predators can coexist in the same area by using resources differently across space and time (Litvaitis and Harrison 1989; Neale and Sacks 2001; Melville et al. 2020). In fact, lagomorph space use was not related to coyote presence, suggesting they may not influence coyote behavior in the study area. Therefore, bobcats and coyotes likely did not compete for the same prey. Further research comparing feeding habits and temporal activity among predators is needed to better understand resource partitioning throughout the day in the SMO.

Our results showed that bobcats synchronized their activity with lagomorphs during the warm-dry and rainy seasons. Bobcats tended to be more active during dawn and dusk, coinciding with periods of peak lagomorph activity. Andrade-Ponce et al. (2022) reported similar findings; however, their temporal overlap values (Δ_1_ = 0.83) were higher than the highest value we obtained (Δ_1_ = 0.69), possibly because we grouped cottontail rabbits and jackrabbits together. Previous studies in arid habitats of Mexico suggest that bobcats primarily synchronize their activity with cottontail rabbits, which are preferred over jackrabbits (López-Vidal et al. 2014; Durán-Antonio et al. 2020; Andrade-Ponce et al. 2022).

The lower temporal overlap observed during the cold-dry season was associated with increased daytime activity in bobcats, likely in response to colder temperatures and reduced prey abundance (Sunquist and Sunquist 2002). Bobcats can shift their activity patterns seasonally for effective thermoregulation, being more active during warmer daylight hours in colder months to conserve energy and maximize hunting opportunities, while avoiding the hottest parts of the day in warmer months to prevent overheating (Sunquist and Sunquist 2002; Rockhill et al. 2013). This adaptive behavior is consistent with their nocturnal activity during the warm-dry season when temperatures in the study site can exceed 30 °C for most of the day. Cathemeral activity during the rainy season may be due to the increased daytime activity of lagomorphs and the heightened hunting activity of females to meet the high energy demands of lactation (Chamberlain et al. 2003; McNitt et al. 2020; Melville et al. 2020). Daytime movements of bobcats facilitate opportunistic predation on tree squirrels and ground squirrels.

Despite our small sample size, our modeling allowed us to evaluate the effects of prey availability on the spatio-temporal patterns of bobcats. Increasing the number of sampling sites could further enhance the predictive power of occupancy modeling by capturing a broader range of environmental effects and species responses, thereby reducing uncertainty in parameter estimates (Mackenzie et al. 2017). In addition, the spatial scale used for the squirrel covariates may not have captured key microhabitats. Using finer scales could enhance model sensitivity to habitat effects. We also were unable to analyze annual changes due to incomplete data from the second year; future studies should extend the sampling period to allow such comparisons. Similarly, we could not evaluate the importance of other small mammal prey using camera traps. Complementary diet analyses and spatial approaches, such as direct trapping, could help assess the role of additional prey including rats, mice, and voles.

Overall, our results showed that bobcat space use and activity were primarily driven by lagomorphs, responding indirectly to habitat by tracking them, as lagomorphs prioritize habitat quality over predation risk. The strength of this relationship varied seasonally, likely in response to climate, energetic demands, and reproductive behavior. During the cold–dry season, bobcats and lagomorphs co-occurred mainly in space; during the warm–dry season, mainly in time; and during the rainy season, under favorable conditions, in both space and time. Weak or negative associations with squirrels are consistent with their role as alternative prey when and where lagomorphs are less abundant (Lariviere and Walton 1997; Aranda et al. 2002; López-Vidal et al. 2014). These patterns highlight the importance of prey in shaping predator space use and emphasize the need to analyze predator–prey interactions across spatial and temporal dimensions simultaneously. Furthermore, the availability of cover and diverse prey in the SMO helps reduce direct competition between bobcats and coyotes.

### Conservation implications

Our results underscore the pivotal role of lagomorphs in shaping the spatio-temporal patterns of bobcats in the SMO. To support this predator–prey dynamic, it is necessary to maintain a mosaic of vegetation within coniferous forests, ensuring suitable cover during the cold-dry months. This habitat structure not only supports lagomorph populations but also provides bobcats with ample opportunities for hunting and concealment. Conservation strategies must also prioritize the preservation of water sources as key areas for ground squirrels. Protecting riparian zones, maintaining natural flow regimes, and minimizing human disturbances near streams are essential to sustain the ecological balance and support both bobcats and their prey.

Finally, we did not find evidence that cattle grazing at the current stocking rates adversely impacts bobcat space use. However, it is crucial to monitor grazing practices closely, as overgrazing could reduce vegetation cover and intensify competition for food resources with lagomorphs, potentially leading to their displacement. We recommend implementing regular monitoring campaigns to inform sustainable land use practices and mitigate potential disturbances, ensuring the long-term viability of both bobcat populations and their prey.

## Supplementary Material

gyag068_Supplementary_Data

## Supplementary data

**Supplementary Data SD1.** Description of covariates included in models of the probability of use (*ψ*) and detection (*P*) for bobcats and their prey.

**Supplementary Data SD2.** Model selection according to expected log predictive density (ELPD), including MacKenzie–Bailey χ^2^ test results for the top-ranked multi-variable models.

**Supplementary Data SD3.** Mean estimates and covariate effects on the detection probability (*P*) for bobcats and their prey across seasons.
