# Supplementary material for: Spatio-temporal habitat use by bobcats and their prey in the northern Sierra Madre Occidental, Chihuahua, Mexico
Source: J Mammal. 2026 Aug 24;107(5):1112–23. doi: 10.1093/jmammal/gyag068 (PMC13722713; doi:10.1093/jmammal/gyag068)
Supplement: Supplementary file 1 [file gyag068_supplementary_data.zip › Supplementary Data SD3.pdf]

Supplementary Data SD3. Mean estimates and covariate effects on the detection probability (*p*) for bobcats and their prey across seasons.

Table S2. Mean estimates of the detection probability (p), standard deviation (SD) and 95% credible intervals from top detection models for bobcat and their prey across seasons in northern Sierra Madre Occidental, Chihuahua, Mexico, 2017–2019. Prey groups included lagomorphs, tree squirrels, and ground squirrels.

Species	Estimate	SD	2.5%	97.5%
Tree squirrel				
Cold-dry 2017	0.28	0.04	0.21	0.35
Warm-dry 2018	0.24	0.04	0.16	0.33
Rainy 2018	0.26	0.04	0.19	0.34
Cold-dry 2018	0.36	0.04	0.27	0.45
Ground squirrel				
Warm-dry 2018	0.15	0.04	0.08	0.23
Rainy 2018	0.24	0.05	0.16	0.34
Lagomorph				
Cold-dry 2017	0.52	0.04	0.43	0.61
Warm-dry 2018	0.23	0.04	0.15	0.32
Rainy 2018	0.12	0.05	0.05	0.24
Cold-dry 2018	0.36	0.05	0.28	0.45
Bobcat				
Cold-dry 2017	0.16	0.04	0.10	0.24
Warm-dry 2018	0.10	0.03	0.06	0.16
Rainy 2018	0.10	0.03	0.05	0.18
Cold-dry 2018	0.16	0.04	0.09	0.23

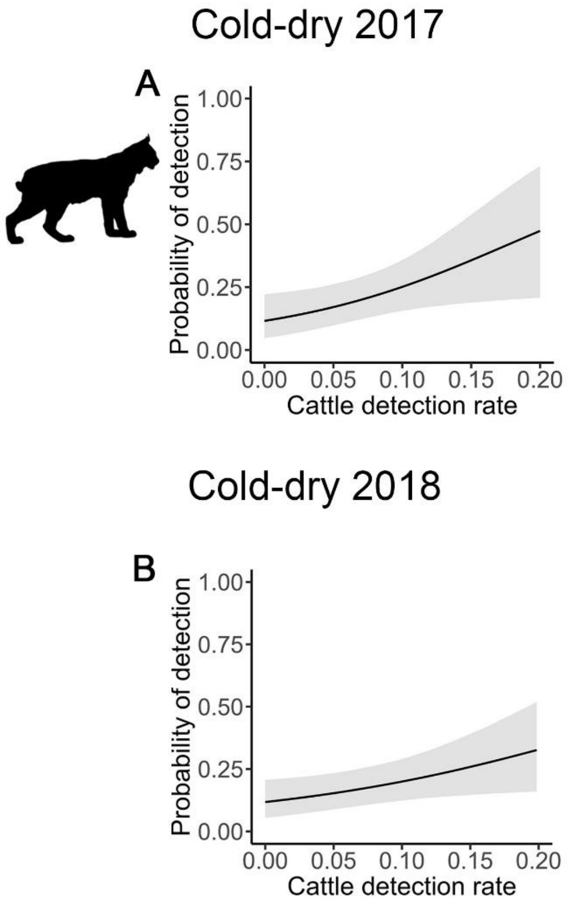

Fig. S1. Covariate effects on the probability of detection (*p*) by bobcats across seasons in northern Sierra Made Occidental, Chihuahua, Mexico, 2017–2019. Only covariates with strong support (95% credible intervals not crossing zero) from the top detection models for the cold-dry 2017 (A) and 2018 (B) seasons are shown. Gray shaded area represents the 95% credible intervals.

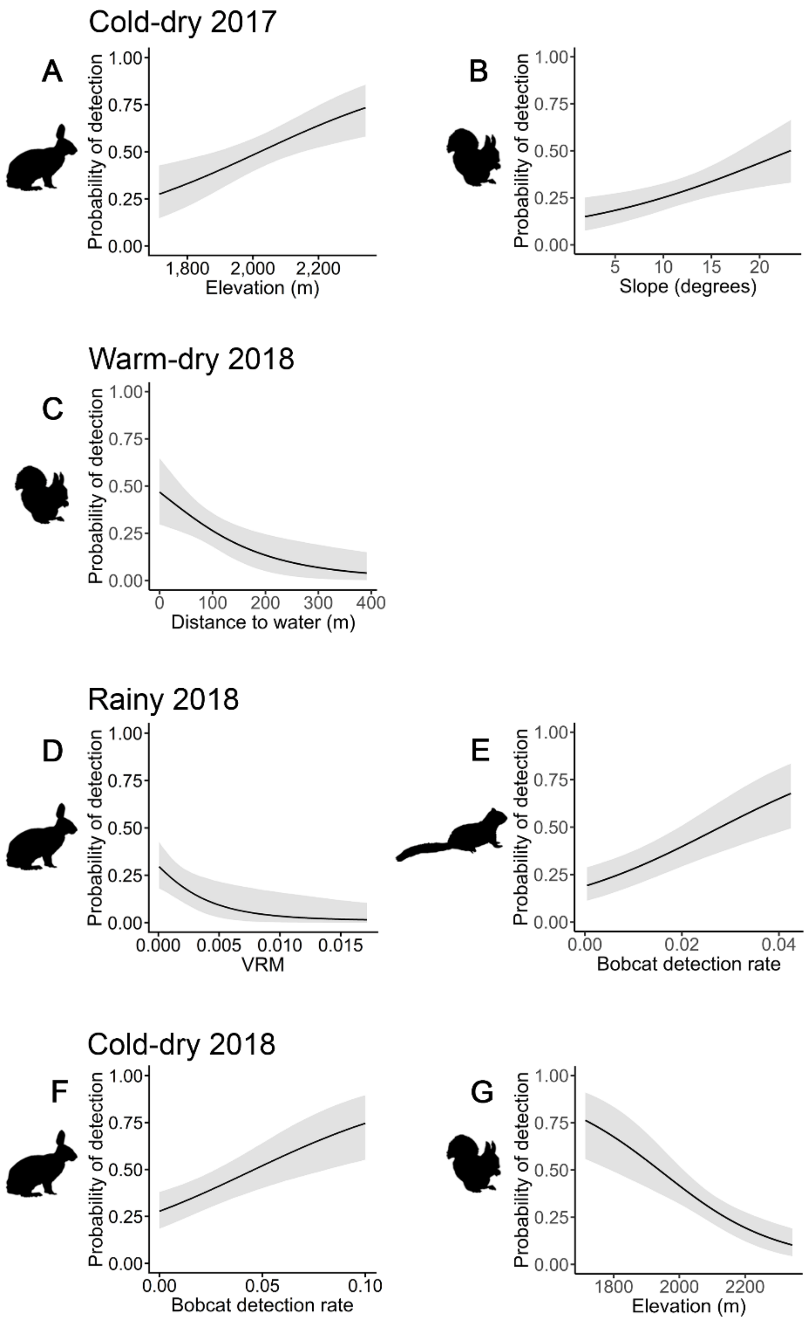

Fig. S2. Covariate effects on detection by prey across seasons in northern Sierra Madre Occidental, Chihuahua, Mexico, 2017–2019. Prey groups included lagomorphs (A, C, D, F), tree squirrels (B), and ground squirrels (E). Only covariates with strong support (95% credible intervals not crossing zero) from the top detection models are shown. Gray shaded area represents the 95% credible intervals.
