# Supplementary material for: Spatio-temporal habitat use by bobcats and their prey in the northern Sierra Madre Occidental, Chihuahua, Mexico
Source: J Mammal. 2026 Aug 24;107(5):1112–23. doi: 10.1093/jmammal/gyag068 (PMC13722713; doi:10.1093/jmammal/gyag068)
Supplement: Supplementary file 1 [file gyag068_supplementary_data.zip › Supplementary Data SD1.pdf]

Supplementary Data SD1. Description of covariates included in models of the probability of use (ψ) and detection (*p*) for bobcats and their prey.

Table S1. Description of covariates used to model probability of use (ψ) and detection (p) of bobcats and their prey in northern Sierra Madre Occidental, Chihuahua, Mexico, 2017–2019. N/A indicates that the variable is not applicable.

**Covariate**	**Description**	**Bobcat**	**Lagomorph**	**Tree squirrel**	**Ground squirrel**
Distance to water	Euclidean distance (m) from each camera site to the nearest water source.	ψ, *p*	ψ, *p*	ψ, *p*	ψ, *p*
Trail	Euclidean distance (m) from each camera site to the nearest trail.	ψ, *p*	ψ, *p*	ψ, *p*	ψ, *p*
Elevation	Mean elevation (m) within a 500 m buffer area for bobcats and 100 m for prey.	ψ, *p*	ψ, *p*	ψ, *p*	ψ, *p*
VRM	Mean vector ruggedness measurement (VRM) within a 500 m buffer area for bobcats and 100 m for prey.	ψ, *p*	ψ, *p*	N/A	ψ, *p*
Slope	Mean slope (degrees) within a buffer of 500 m for bobcats and 100 m for prey.	ψ, *p*	N/A	ψ, *p*	N/A
Canopy cover	Percent of tree canopy cover within a buffer area of 500 m for bobcats and 100 m for prey.	ψ	ψ	ψ	ψ
NDVI	Mean normalized difference vegetation index (NDVI) during each season within a buffer area of 500 m for bobcats and 100 m for prey.	ψ, *p*	ψ, *p*	ψ, *p*	ψ, *p*
Coniferous forest	Percent of coniferous forest cover within a buffer area of 500 m for bobcats and 100 m for prey.	ψ	ψ	ψ	ψ
Deciduous forest	Percent of deciduous forest cover within a buffer area of 500 m for bobcats and 100 m for prey.	ψ	ψ	ψ	ψ
Cattle detection rate	Number of independent detections of cattle at each camera site divided by the number of camera nights during each season.	*p*	*p*	*p*	*p*

Coyote detection rate	Number of independent detections of coyotes at each camera site divided by the number of camera nights during each season.	*p*	*p*	*p*	*p*
Bobcat detection rate	Number of independent detections of bobcats at each camera site divided by the number of camera nights during each season.	N/A	*p*	*p*	*p*
Cattle occurrence	Conditional occupancy of cattle during each season.	ψ	ψ	ψ	ψ
Coyote occurrence	Conditional occupancy of coyotes during each season.	ψ	ψ	ψ	ψ
Bobcat occurrence	Conditional occupancy of bobcats during each season.	N/A	ψ	ψ	ψ
Lagomorph occurrence	Conditional occupancy of lagomorphs during each season.	ψ	N/A	N/A	N/A
Tree squirrel occurrence	Conditional occupancy of tree squirrels during each season.	ψ	N/A	N/A	N/A
Ground squirrel occurrence	Conditional occupancy of ground squirrels during each season.	ψ	N/A	N/A	N/A
