# Supplementary material for: Spatio-temporal habitat use by bobcats and their prey in the northern Sierra Madre Occidental, Chihuahua, Mexico
Source: J Mammal. 2026 Aug 24;107(5):1112–23. doi: 10.1093/jmammal/gyag068 (PMC13722713; doi:10.1093/jmammal/gyag068)
Supplement: Supplementary file 1 [file gyag068_supplementary_data.zip › Supplementary Data SD2.pdf]

Supplementary Data SD2 — Model selection according to expected log predictive density (ELPD), including MacKenzie–Bailey χ^2 test results for the top-ranked multi-variable models.

Bobcat

Cold-dry 2017

Model	elpd	nparam	elpd_diff	se_diff	weight	χ^2	*P* value
Detection							
ψ(.),p(Cattle_DR)	-77.06	2.99	0.00	0.00	0.84		
ψ(.),p(Coyote_DR)	-78.18	4.29	-1.12	1.78	0.11		
ψ(.),p(Trail)	-78.87	2.98	-1.81	2.15	0.05		
ψ(.),p(.)	-79.18	2.70	-2.12	1.83	0.00		
ψ(.),p(Elevation)	-79.69	3.24	-2.63	1.81	0.00		
ψ(.),p(Water)	-80.30	3.87	-3.24	2.26	0.00		
ψ(.),p(NDVI)	-80.55	4.05	-3.49	2.10	0.00		
ψ(.),p(VRM)	-81.66	5.37	-4.59	3.05	0.00		
ψ(.),p(Slope)	-81.85	6.02	-4.79	2.59	0.00		
Univariate occupancy							
ψ(Deciduous),p(Cattle_DR)	-74.08	3.48	0.00	0.00	0.91		
ψ(Coniferous),p(Cattle_DR)	-74.99	3.65	-0.91	0.59	0.00		
ψ(.),p(Cattle_DR)	-77.06	2.99	-2.98	2.26	0.09		
ψ(Slope),p(Cattle_DR)	-77.49	4.20	-3.41	1.94	0.00		
ψ(VRM),p(Cattle_DR)	-77.65	4.33	-3.57	2.34	0.00		
ψ(Canopy),p(Cattle_DR)	-77.82	4.22	-3.74	2.43	0.00		
ψ(Water),p(Cattle_DR)	-77.89	4.02	-3.81	2.29	0.00		
ψ(NDVI),p(Cattle_DR)	-78.05	4.19	-3.97	2.35	0.00		
(Elevation),p(Cattle_DR)	-78.21	4.29	-4.13	2.31	0.00		
Multi-variable occupancy							
ψ(Deciduous+Tree squirrel+Coyote), p(Cattle_DR)	-71.54	4.45	0.00	0.00	0.31	2107.62	0.35
ψ(Deciduous+Lagomorph+NDVI), p(Cattle_DR)	-72.21	4.45	-0.67	2.33	0.45	2808.05	0.30
ψ(Deciduous+Tree squirrel+Trail), p(Cattle_DR)	-72.37	4.07	-0.83	1.37	0.09	2383.35	0.32
ψ(Deciduous+Lagomorph+Tree squirrel), p(Cattle_DR)	-72.52	4.34	-0.98	1.71	0.00		
ψ(Deciduous+Tree squirrel), p(Cattle_DR)	-73.03	3.96	-1.48	1.32	0.00		
ψ(Deciduous+Lagomorph), p(Cattle_DR)	-73.14	3.97	-1.59	2.01	0.00		
ψ(Deciduous+Tree squirrel+Canopy), p(Cattle_DR)	-73.59	4.66	-2.05	1.57	0.00		
ψ(Deciduous+Canopy+NDVI), p(Cattle_DR)	-73.94	4.50	-2.39	2.33	0.12		
ψ(Deciduous+Lagomorph+Cattle), p(Cattle_DR)	-73.96	4.88	-2.41	2.18	0.00		
ψ(Deciduous+Canopy), p(Cattle_DR)	-74.04	4.03	-2.49	2.06	0.00		
ψ(Deciduous),p(Cattle_DR)	-74.13	3.53	-2.58	1.90	0.02		
ψ(Deciduous+Lagomorph+Trail), p(Cattle_DR)	-74.14	5.00	-2.60	2.26	0.00		
ψ(Deciduous+Lagomorph+Coyote), p(Cattle_DR)	-74.19	5.71	-2.65	2.40	0.00		
ψ(Deciduous+Elevation), p(Cattle_DR)	-74.49	4.27	-2.95	2.11	0.00		
ψ(Deciduous+NDVI), p(Cattle_DR)	-74.52	4.24	-2.98	2.20	0.00		
ψ(Deciduous+Cattle), p(Cattle_DR)	-74.70	4.18	-3.15	1.93	0.00		
ψ(Deciduous+Coyote), p(Cattle_DR)	-74.71	4.85	-3.17	1.75	0.00		
ψ(Deciduous+Coyote+Canopy), p(Cattle_DR)	-74.87	5.47	-3.33	1.98	0.00		
ψ(Deciduous+Cattle+NDVI), p(Cattle_DR)	-75.15	4.95	-3.61	2.21	0.00		

Warm-dry 2018

Model	elpd	nparam	elpd_diff	se_diff	weight	χ^2	*P* value
Detection							
ψ(.),p(.)	-68.38	2.00	0.00	0.00	0.67		
ψ(.),p(Coyote_DR)	-68.63	3.17	-0.25	1.22	0.33		
ψ(.),p(Water)	-69.23	2.86	-0.84	0.72	0.00		
ψ(.),p(Trail)	-69.32	3.13	-0.94	0.55	0.00		
ψ(.),p(VRM)	-69.36	2.85	-0.98	0.32	0.00		
ψ(.),p(NDVI)	-69.50	2.94	-1.11	0.32	0.00		
ψ(.),p(Cattle_DR)	-69.57	3.13	-1.19	0.88	0.00		
ψ(.),p(Elevation)	-69.69	4.07	-1.31	1.02	0.00		
ψ(.),p(Slope)	-69.74	3.43	-1.36	0.52	0.00		
Univariate occupancy							
ψ(Canopy),p(.)	-67.77	2.53	0.00	0.00	0.90		
ψ(.),p(.)	-68.38	2.00	-0.61	0.96	0.00		
ψ(NDVI),p(.)	-68.58	2.52	-0.81	1.25	0.10		
ψ(Coniferous),p(.)	-68.68	2.80	-0.91	0.96	0.00		
ψ(Slope),p(.)	-68.68	2.58	-0.91	1.24	0.00		
ψ(VRM),p(.)	-68.79	2.51	-1.02	1.18	0.00		
ψ(Water),p(.)	-68.83	2.84	-1.05	1.22	0.00		
ψ(Elevation),p(.)	-68.88	2.68	-1.11	1.25	0.00		
ψ(Deciduous),p(.)	-68.94	2.83	-1.17	0.97	0.00		
Multi-variable occupancy							
ψ(Canopy+Lagomorph+Ground Squirrel),p(.)	-65.95	3.29	0.00	0.00	0.41	1979.10	0.27
ψ(Canopy+Lagomorph+Tree Squirrel),p(.)	-67.25	3.94	-1.30	1.47	0.13	2045.72	0.27
ψ(Canopy+Ground Squirrel+Cattle),p(.)	-67.46	3.31	-1.50	1.40	0.24		
ψ(Canopy+Lagomorph+NDVI),p(.)	-67.56	4.02	-1.61	1.24	0.00		
ψ(Canopy),p(.)	-67.77	2.53	-1.82	1.26	0.13		
ψ(Canopy+Ground Squirrel),p(.)	-67.78	2.98	-1.83	1.31	0.00		
ψ(Canopy+Lagomorph),p(.)	-67.81	3.49	-1.86	1.06	0.00		
ψ(Canopy+Cattle),p(.)	-67.81	2.97	-1.86	1.30	0.00		
ψ(Canopy+NDVI),p(.)	-68.02	3.29	-2.07	1.45	0.06		
ψ(Canopy+VRM),p(.)	-68.10	3.08	-2.15	1.40	0.02		
ψ(Canopy+Cattle+NDVI),p(.)	-68.13	3.67	-2.18	1.48	0.00		
ψ(Canopy+Ground Squirrel+Coyote),p(.)	-68.25	3.58	-2.30	1.37	0.00		
ψ(Canopy+Ground Squirrel+Trail),p(.)	-68.31	3.60	-2.36	1.57	0.00		
ψ(Canopy+Coyote),p(.)	-68.32	3.14	-2.37	1.35	0.00		
ψ(Canopy+Water),p(.)	-68.34	3.26	-2.39	1.27	0.00		
ψ(Canopy+Lagomorph+Trail),p(.)	-68.40	4.25	-2.44	1.13	0.00		
ψ(Canopy+Ground Squirrel+VRM),p(.)	-68.44	3.69	-2.48	1.40	0.00		
ψ(Canopy+Tree Squirrel),p(.)	-68.44	3.27	-2.49	1.32	0.00		
ψ(Canopy+Lagomorph+Cattle),p(.)	-68.52	4.30	-2.57	1.12	0.00		
ψ(Canopy+NDVI+VRM),p(.)	-68.53	4.03	-2.58	1.76	0.00		
ψ(Canopy+NDVI+Water),p(.)	-68.56	4.01	-2.61	1.43	0.00		
ψ(Canopy+Lagomorph+Coyote),p(.)	-68.62	4.39	-2.67	1.34	0.00		
ψ(Canopy+Tree Squirrel+Trail),p(.)	-68.69	3.76	-2.74	1.49	0.00		
ψ(Canopy+Tree Squirrel+Coyote),p(.)	-68.84	3.80	-2.89	1.41	0.00		

Rainy 2018

Model	elpd	nparam	elpd_diff	se_diff	weight	χ^2	*P* value
Detection							
ψ(.),p(.)	-49.83	1.80	0.00	0.00	0.92		
ψ(.),p(NDVI)	-50.13	2.63	-0.30	0.86	0.08		
ψ(.),p(Elevation)	-50.28	2.36	-0.45	0.52	0.00		
ψ(.),p(Trail)	-50.43	2.29	-0.60	0.48	0.00		
ψ(.),p(Slope)	-50.45	2.67	-0.62	0.68	0.00		
ψ(.),p(Water)	-50.49	2.60	-0.66	1.06	0.00		
ψ(.),p(Coyote_DR)	-50.74	2.88	-0.91	0.67	0.00		
ψ(.),p(Cattle_DR)	-50.76	2.70	-0.93	0.43	0.00		
ψ(.),p(VRM)	-51.02	2.80	-1.19	0.67	0.00		
Univariate occupancy							
ψ(.),p(.)	-49.83	1.80	0.00	0.00	0.98		
ψ(NDVI),p(.)	-50.39	2.88	-0.56	0.99	0.02		
ψ(Slope),p(.)	-50.66	2.84	-0.83	0.65	0.00		
ψ(Canopy),p(.)	-50.72	2.80	-0.89	0.51	0.00		
ψ(Coniferous),p(.)	-50.80	2.87	-0.97	0.55	0.00		
ψ(Deciduous),p(.)	-50.95	3.00	-1.12	0.62	0.00		
ψ(Elevation),p(.)	-51.07	3.09	-1.24	0.61	0.00		
ψ(VRM),p(.)	-51.07	3.00	-1.24	0.49	0.00		
ψ(Water),p(.)	-51.08	3.02	-1.25	0.59	0.00		
Multi-variable occupancy							
ψ(Lagomorph+Ground Squirrel),p(.)	-47.10	2.64	0.00	0.00	0.26	304.58	0.48
ψ(Lagomorph+Tree Squirrel),p(.)	-47.63	2.63	-0.53	1.04	0.17	317.09	0.46
ψ(Lagomorph),p(.)	-48.34	2.23	-1.25	1.04	0.00		
ψ(Lagomorph+NDVI),p(.)	-48.79	3.17	-1.69	1.16	0.00		
ψ(Lagomorph+Cattle),p(.)	-49.09	3.56	-2.00	2.15	0.00		
ψ(Cattle+NDVI),p(.)	-49.28	3.68	-2.18	2.49	0.44	353.72	0.47
ψ(Cattle),p(.)	-49.40	2.82	-2.30	2.37	0.04	355.99	0.49
ψ(Lagomorph+Coyote),p(.)	-49.45	3.42	-2.36	1.42	0.00		
ψ(Ground Squirrel+Cattle),p(.)	-49.78	3.74	-2.69	2.25	0.00		
ψ(.),p(.)	-49.83	1.80	-2.73	1.63	0.08		
ψ(Tree Squirrel+Canopy),p(.)	-50.50	3.34	-3.41	1.78	0.00		
ψ(Tree Squirrel),p(.)	-50.58	2.84	-3.49	2.05	0.00		
ψ(Canopy+NDVI),p(.)	-50.69	3.45	-3.59	1.76	0.00		
ψ(Ground Squirrel),p(.)	-50.73	2.95	-3.63	1.78	0.00		
ψ(Coyote),p(.)	-50.88	2.87	-3.79	1.63	0.00		
ψ(Slope+NDVI),p(.)	-50.96	3.46	-3.86	1.99	0.00		
ψ(Ground Squirrel+Coyote),p(.)	-51.29	3.66	-4.19	1.63	0.00		
ψ(VRM+NDVI),p(.)	-51.29	3.84	-4.20	1.99	0.00		
ψ(Coyote+Canopy),p(.)	-51.58	3.67	-4.49	1.53	0.00		
ψ(Tree Squirrel+Trail),p(.)	-51.60	3.79	-4.50	1.96	0.00		
ψ(Tree Squirrel+Coyote),p(.)	-51.70	4.01	-4.60	2.27	0.00		
ψ(Ground Squirrel+VRM),p(.)	-51.86	4.03	-4.76	1.72	0.00		
ψ(VRM+Canopy),p(.)	-51.93	3.98	-4.83	1.72	0.00		
ψ(Ground Squirrel+Trail),p(.)	-52.38	4.57	-5.28	2.12	0.00		

Cold-dry 2018

Model	elpd	nparam	elpd_diff	se_diff	weight	χ^2	*P* value
Detection							
ψ(.),p(Cattle_DR)	-76.36	2.49	0.00	0.00	0.58		
ψ(.),p(Coyote_DR)	-77.09	3.32	-0.73	1.84	0.22		
ψ(.),p(Trail)	-77.31	2.75	-0.96	2.06	0.13		
ψ(.),p(.)	-78.04	2.27	-1.68	1.69	0.00		
ψ(.),p(VRM)	-78.12	3.21	-1.76	2.63	0.07		
ψ(.),p(Slope)	-79.03	4.13	-2.68	3.31	0.00		
ψ(.),p(Elevation)	-79.06	3.58	-2.70	1.83	0.00		
ψ(.),p(Water)	-79.09	3.28	-2.74	1.97	0.00		
ψ(.),p(NDVI)	-79.49	3.78	-3.13	2.13	0.00		
Univariate occupancy							
ψ(Water),p(Cattle_DR)	-75.86	3.20	0.00	0.00	0.49		
ψ(Deciduous),p(Cattle_DR)	-76.22	3.80	-0.36	2.53	0.50		
ψ(.),p(Cattle_DR)	-76.36	2.49	-0.50	1.30	0.00		
ψ(Coniferous),p(Cattle_DR)	-76.58	3.81	-0.72	2.22	0.00		
ψ(Canopy),p(Cattle_DR)	-76.60	3.37	-0.74	1.60	0.00		
ψ(NDVI),p(Cattle_DR)	-76.73	3.57	-0.87	1.86	0.01		
ψ(Slope),p(Cattle_DR)	-76.91	3.53	-1.05	1.60	0.00		
ψ(VRM),p(Cattle_DR)	-77.43	3.80	-1.57	1.68	0.00		
ψ(Elevation),p(Cattle_DR)	-77.46	3.52	-1.60	1.45	0.00		
Multi-variable occupancy							
ψ(Water+Lagomorph), p(Cattle_DR)	-75.59	3.80	0.00	0.00	0.60	876.31	0.58
ψ(Water), p(Cattle_DR)	-75.92	3.26	-0.33	1.16	0.22	795.36	0.59
ψ(Water+Lagomorph+NDVI), p(Cattle_DR)	-76.40	4.75	-0.81	0.65	0.00		
ψ(Water+NDVI), p(Cattle_DR)	-76.44	4.19	-0.86	1.23	0.00		
ψ(Water+Canopy), p(Cattle_DR)	-76.58	4.53	-0.99	1.93	0.10	794.35	0.59
ψ(Water+Lagomorph+Tree squirrel), p(Cattle_DR)	-76.61	4.80	-1.02	0.46	0.00		
ψ(Water+Lagomorph+Coyote), p(Cattle_DR)	-76.68	5.27	-1.09	1.36	0.00		
ψ(Water+Elevation), p(Cattle_DR)	-76.70	4.01	-1.11	1.21	0.00		
ψ(Water+Tree squirrel), p(Cattle_DR)	-76.75	4.31	-1.17	1.04	0.00		
ψ(Water+Lagomorph+Cattle), p(Cattle_DR)	-76.78	5.65	-1.19	2.02	0.00		
ψ(Water+Coyote+Canopy), p(Cattle_DR)	-76.87	5.45	-1.29	1.91	0.04	789.94	0.56
ψ(Water+Canopy+NDVI), p(Cattle_DR)	-77.00	5.33	-1.42	1.80	0.00		
ψ(Water+Cattle), p(Cattle_DR)	-77.23	5.06	-1.64	2.34	0.00		
ψ(Water+Coyote), p(Cattle_DR)	-77.25	4.89	-1.66	1.86	0.00		
ψ(Water+Tree squirrel+Trail), p(Cattle_DR)	-77.49	5.03	-1.91	1.08	0.00		
ψ(Water+Cattle+NDVI), p(Cattle_DR)	-77.56	6.08	-1.98	2.59	0.05	806.86	0.57
ψ(Water+Tree squirrel+Coyote), p(Cattle_DR)	-77.99	5.77	-2.40	1.68	0.00		

Lagomorpha

Cold-dry 2017

Model	elpd	nparam	elpd_diff	se_diff	weight	χ2	*P* value
Detection							
ψ(.),p(Elevation)	-115.31	4.56	0.00	0.00	0.39		
ψ(.),p(Trail)	-115.45	5.09	-0.14	4.70	0.40		
ψ(.),p(Coyote_DR)	-118.65	4.71	-3.34	4.80	0.00		
ψ(.),p(Bobcat_DR)	-118.73	4.38	-3.42	5.56	0.21		
ψ(.),p(NDVI)	-119.09	6.10	-3.78	4.07	0.00		
ψ(.),p(.)	-119.16	3.34	-3.85	4.85	0.00		
ψ(.),p(Cattle_DR)	-119.56	5.55	-4.24	3.76	0.00		
ψ(.),p(Water)	-121.00	6.10	-5.69	5.90	0.00		
ψ(.),p(Slope)	-121.35	6.90	-6.03	6.02	0.00		
Univariate occupancy							
ψ(VRM),p(Elevation)	-105.31	4.63	0.00	0.00	0.89		
ψ(Deciduous),p(Elevation)	-113.77	5.49	-8.46	2.61	0.00		
ψ(Coniferous),p(Elevation)	-113.89	5.50	-8.57	2.87	0.11		
ψ(Water),p(Elevation)	-114.98	5.56	-9.66	2.57	0.00		
ψ(.),p(Elevation)	-115.31	4.56	-10.00	2.23	0.00		
ψ(Elevation),p(Elevation)	-115.98	5.66	-10.67	2.63	0.00		
ψ(NDVI),p(Elevation)	-116.44	5.73	-11.12	2.31	0.00		
ψ(Canopy),p(Elevation)	-116.49	5.75	-11.18	2.25	0.00		
Multi-variable occupancy							
ψ(VRM+Bobcat+Coyote),p(Elevation)	-102.79	5.52	0.00	0.00	0.56	956.63	0.72
ψ(VRM+Bobcat),p(Elevation)	-104.68	5.35	-1.89	1.13	0.00		
ψ(VRM+Bobcat+NDVI),p(Elevation)	-105.20	5.91	-2.41	1.36	0.00		
ψ(VRM),p(Elevation)	-105.31	4.63	-2.52	1.46	0.31		
ψ(VRM+Bobcat+Trail),p(Elevation)	-105.35	6.07	-2.56	1.32	0.00		
ψ(VRM+Elevation),p(Elevation)	-105.48	5.27	-2.69	1.63	0.04		
ψ(VRM+Coyote),p(Elevation)	-105.57	5.22	-2.78	1.54	0.00		
ψ(VRM+Canopy),p(Elevation)	-105.68	5.30	-2.89	1.51	0.00		
ψ(VRM+Canopy+Elevation),p(Elevation)	-105.91	6.26	-3.12	2.08	0.10		
ψ(VRM+NDVI),p(Elevation)	-106.21	5.46	-3.42	1.67	0.00		
ψ(VRM+Cattle),p(Elevation)	-106.28	5.56	-3.49	1.70	0.00		
ψ(VRM+Coyote+Canopy),p(Elevation)	-106.39	6.31	-3.60	1.87	0.00		
ψ(VRM+Coyote+Trail),p(Elevation)	-106.48	6.25	-3.69	1.71	0.00		
ψ(VRM+Canopy+NDVI),p(Elevation)	-106.59	6.18	-3.80	1.68	0.00		
ψ(VRM+Cattle+NDVI),p(Elevation)	-107.18	6.42	-4.39	2.05	0.00		

Warm-dry 2018

Model	elpd	nparam	elpd_diff	se_diff	weight	χ2	*P* value
Detection							
ψ(.),p(Coyote_DR)	-81.93	1.94	0.00	0.00	0.52		
ψ(.),p(VRM)	-82.01	2.62	-0.09	1.40	0.48		
ψ(.),p(.)	-82.26	1.58	-0.33	0.77	0.00		
ψ(.),p(Trail)	-82.93	2.08	-1.00	1.09	0.00		
ψ(.),p(Cattle_DR)	-82.97	2.27	-1.04	0.76	0.00		
ψ(.),p(Elevation)	-82.98	2.04	-1.05	0.84	0.00		
ψ(.),p(NDVI)	-83.12	2.15	-1.19	0.86	0.00		
ψ(.),p(Water)	-83.14	2.18	-1.21	0.80	0.00		
ψ(.),p(Bobcat_DR)	-83.35	2.48	-1.42	0.91	0.00		
Univariate occupancy							
ψ(VRM),p(Coyote_DR)	-80.61	2.87	0.00	0.00	0.77		
ψ(.),p(Coyote_DR)	-81.93	1.94	-1.32	2.14	0.00		
ψ(Water),p(Coyote_DR)	-81.98	3.02	-1.37	2.46	0.23		
ψ(Deciduous),p(Coyote_DR)	-82.65	3.27	-2.03	1.57	0.00		
ψ(Coniferous),p(Coyote_DR)	-82.82	3.21	-2.21	1.66	0.00		
ψ(Canopy),p(Coyote_DR)	-82.87	3.10	-2.25	2.34	0.00		
ψ(Elevation),p(Coyote_DR)	-83.00	3.12	-2.38	2.35	0.00		
ψ(NDVI),p(Coyote_DR)	-83.21	3.24	-2.60	2.25	0.00		
Multi-variable occupancy							
ψ(VRM),p(Coyote)	-80.61	2.87	0.00	0.00	0.09	650.02	0.64
ψ(VRM+Bobcat),p(Coyote_DR)	-80.78	4.23	-0.16	1.89	0.41	687.95	0.63
ψ(VRM+Canopy),p(Coyote_DR)	-80.94	3.98	-0.32	1.34	0.42	686.44	0.66
ψ(VRM+Water),p(Coyote_DR)	-81.31	3.95	-0.70	1.20	0.08	694.23	0.65
ψ(VRM+Cattle),p(Coyote_DR)	-81.45	4.17	-0.84	1.26	0.00		
ψ(VRM+Bobcat+Coyote),p(Coyote_DR)	-81.56	5.08	-0.95	2.08	0.00		
ψ(VRM+Coyote),p(Coyote_DR)	-81.65	3.91	-1.04	0.42	0.00		
ψ(VRM+Canopy+Water),p(Coyote_DR)	-81.75	5.22	-1.13	1.71	0.00		
ψ(VRM+NDVI),p(Coyote_DR)	-81.94	4.20	-1.33	0.48	0.00		
ψ(VRM+Bobcat+Trail),p(Coyote_DR)	-81.97	5.66	-1.36	1.96	0.00		
ψ(VRM+Coyote+Trail),p(Coyote_DR)	-81.97	5.20	-1.36	1.41	0.00		
ψ(VRM+Canopy+NDVI),p(Coyote_DR)	-81.97	5.05	-1.36	1.32	0.00		
ψ(VRM+Bobcat+NDVI),p(Coyote_DR)	-82.23	5.61	-1.62	1.96	0.00		
ψ(VRM+Water+NDVI),p(Coyote_DR)	-82.78	5.43	-2.17	1.36	0.00		
ψ(VRM+Cattle+NDVI),p(Coyote_DR)	-82.87	5.58	-2.26	1.34	0.00		
ψ(VRM+Coyote+Canopy),p(Coyote_DR)	-83.36	5.75	-2.75	1.09	0.00		

Rainy 2018

Model	elpd	nparam	elpd_diff	se_diff	weight	χ2	*P* value
Detection							
ψ(.),p(VRM)	-68.04	4.26	0.00	0.00	0.78		
ψ(.),p(NDVI)	-69.14	3.56	-1.10	2.32	0.00		
ψ(.),p(.)	-70.45	2.45	-2.42	3.75	0.00		
ψ(.),p(Cattle_DR)	-70.97	3.33	-2.93	3.80	0.00		
ψ(.),p(Elevation)	-71.03	3.13	-2.99	3.14	0.00		
ψ(.),p(Trail)	-71.43	4.45	-3.39	4.51	0.22		
ψ(.),p(Water)	-71.72	3.66	-3.68	3.70	0.00		
ψ(.),p(Coyote_DR)	-71.89	3.83	-3.85	3.73	0.00		
ψ(.),p(Bobcat_DR)	-72.71	5.14	-4.67	4.14	0.00		
Univariate occupancy							
ψ(Water),p(VRM)	-66.25	4.38	0.00	0.00	0.54		
ψ(VRM),p(VRM)	-66.37	4.40	-0.12	2.61	0.46		
ψ(.),p(VRM)	-68.04	4.26	-1.79	1.59	0.00		
ψ(NDVI),p(VRM)	-68.24	5.11	-1.99	1.95	0.00		
ψ(Deciduous),p(VRM)	-68.31	5.54	-2.06	2.05	0.00		
ψ(Canopy),p(VRM)	-68.43	5.23	-2.18	1.76	0.00		
ψ(Coniferous),p(VRM)	-68.44	5.43	-2.19	1.79	0.00		
ψ(Elevation),p(VRM)	-69.18	5.56	-2.94	1.61	0.00		
Multi-variable occupancy							
ψ(Water+Bobcat),p(VRM)	-62.74	4.14	0.00	0.00	0.04	1433.38	0.45
ψ(Water+Bobcat+Trail),p(VRM)	-62.79	4.77	-0.05	0.80	0.25	1407.90	0.44
ψ(Water+Bobcat+NDVI),p(VRM)	-63.20	5.01	-0.46	0.94	0.23	1365.31	0.46
ψ(Water+Bobcat+Coyote),p(VRM)	-63.73	5.40	-0.99	1.05	0.13	1403.92	0.45
ψ(Water+VRM),p(VRM)	-65.63	4.96	-2.89	2.57	0.36		
ψ(Water+Cattle),p(VRM)	-65.97	5.06	-3.23	2.21	0.00		
ψ(Water),p(VRM)	-66.25	4.38	-3.51	2.28	0.00		
ψ(Water+VRM+NDVI),p(VRM)	-66.42	5.83	-3.68	2.83	0.00		
ψ(Water+Canopy),p(VRM)	-66.48	5.20	-3.74	2.38	0.00		
ψ(Water+NDVI),p(VRM)	-67.08	5.51	-4.34	2.68	0.00		
ψ(Water+Cattle+NDVI),p(VRM)	-67.08	6.18	-4.34	2.56	0.00		
ψ(Water+Coyote),p(VRM)	-67.23	5.71	-4.49	2.91	0.00		
ψ(Water+Canopy+NDVI),p(VRM)	-67.26	6.15	-4.53	2.59	0.00		
ψ(Water+Coyote+Canopy),p(VRM)	-67.85	6.94	-5.11	2.94	0.00		
ψ(Water+Coyote+Trail),p(VRM)	-68.28	6.94	-5.54	3.19	0.00		

Cold-dry 2018

Model	elpd	nparam	elpd_diff	se_diff	weight	χ2	*P* value
Detection							
ψ(.),p(Bobcat_DR)	-101.59	7.42	0.00	0.00	0.44		
ψ(.),p(VRM)	-104.40	9.03	-2.80	7.38	0.40		
ψ(.),p(Elevation)	-104.53	7.36	-2.94	8.13	0.16		
ψ(.),p(.)	-106.67	5.54	-5.07	7.17	0.00		
ψ(.),p(NDVI)	-107.13	8.54	-5.54	8.08	0.00		
ψ(.),p(Cattle_DR)	-108.67	11.10	-7.08	7.71	0.00		
ψ(.),p(Trail)	-108.82	9.81	-7.22	7.97	0.00		
ψ(.),p(water)	-109.53	9.83	-7.93	7.39	0.00		
ψ(.),p(Coyote_DR)	-109.99	10.13	-8.39	9.31	0.00		
Univariate occupancy							
ψ(VRM),p(Bobcat_DR)	-95.65	7.58	0.00	0.00	0.79		
ψ(NDVI),p(Bobcat_DR)	-101.64	8.24	-6.00	3.10	0.00		
ψ(Deciduous),p(Bobcat_DR)	-102.04	8.49	-6.40	2.89	0.00		
ψ(Water),p(Bobcat_DR)	-102.14	8.11	-6.50	2.53	0.00		
ψ(Elevation),p(Bobcat_DR)	-102.36	8.40	-6.72	2.74	0.00		
ψ(Canopy),p(Bobcat_DR)	-102.45	8.35	-6.81	2.74	0.00		
ψ(Coniferous),p(Bobcat_DR)	-102.49	8.50	-6.85	2.73	0.00		
ψ(.),p(Bobcat_DR)	-104.40	9.03	-8.75	7.31	0.21		
Multi-variable occupancy							
ψ(Water+Bobcat),p(Bobcat_DR)	-94.83	7.99	0.00	0.00	0.43	962.20	0.75
ψ(VRM+Bobcat+NDVI),p(Bobcat_DR)	-95.03	8.68	-0.21	0.87	0.21	983.26	0.73
ψ(VRM+NDVI),p(Bobcat_DR)	-95.42	8.02	-0.60	1.35	0.00		
ψ(VRM),p(Bobcat_DR)	-95.65	7.58	-0.82	1.22	0.00		
ψ(VRM+Bobcat+Trail),p(Bobcat_DR)	-95.74	8.94	-0.91	0.38	0.00		
ψ(VRM+Cattle),p(Bobcat_DR)	-95.83	8.67	-1.01	2.07	0.29	841.50	0.81
ψ(VRM+Canopy),p(Bobcat_DR)	-95.83	8.20	-1.01	1.61	0.07	868.05	0.79
ψ(VRM+Cattle+NDVI),p(Bobcat_DR)	-95.95	8.89	-1.13	2.09	0.00		
ψ(VRM+Canopy+NDVI),p(Bobcat_DR)	-96.06	8.95	-1.23	1.59	0.00		
ψ(VRM+Bobcat+Coyote),p(Bobcat_DR)	-96.20	9.43	-1.38	0.62	0.00		
ψ(VRM+Elevation),p(Bobcat_DR)	-96.61	8.82	-1.79	1.54	0.00		
ψ(VRM+Canopy+Elevation),p(Bobcat_DR)	-96.85	9.38	-2.03	1.76	0.00		
ψ(VRM+Coyote),p(Bobcat_DR)	-96.89	8.91	-2.07	1.63	0.00		
ψ(VRM+Coyote+Canopy),p(Bobcat_DR)	-97.22	9.52	-2.40	1.83	0.00		
ψ(VRM+Coyote+Trail),p(Bobcat_DR)	-98.05	10.08	-3.22	1.91	0.00		

Tree Squirrel

Cold-dry 2017

Model	elpd	nparam	elpd_diff	se_diff	weight	χ2	P value
Detection							
ψ(.),p(Slope)	-126.25	4.08	0.00	0.00	0.66		
ψ(.),p(Bobcat_DR)	-128.58	3.57	-2.33	3.79	0.32		
ψ(.),p(NDVI)	-129.16	4.74	-2.91	3.24	0.00		
ψ(.),p(.)	-129.29	2.92	-3.04	4.06	0.00		
ψ(.),p(Coyote_DR)	-129.50	3.84	-3.25	4.20	0.03		
ψ(.),p(Water)	-129.66	3.65	-3.40	3.98	0.00		
ψ(.),p(Cattle_DR)	-130.04	6.13	-3.79	3.16	0.00		
ψ(.),p(Trail)	-130.50	5.19	-4.24	4.03	0.00		
ψ(.),p(Elevation)	-131.92	6.41	-5.66	5.02	0.00		
Univariate occupancy							
ψ(.),p(Slope)	-126.25	4.08	0.00	0.00	0.58		
ψ(Slope),p(Slope)	-126.28	5.04	-0.02	1.29	0.42		
ψ(Coniferous),p(Slope)	-127.07	5.23	-0.81	0.89	0.00		
ψ(Deciduous),p(Slope)	-127.20	5.33	-0.95	0.85	0.00		
ψ(Water),p(Slope)	-127.37	5.23	-1.12	0.84	0.00		
ψ(Canopy),p(Slope)	-127.45	5.36	-1.20	0.56	0.00		
ψ(NDVI),p(Slope)	-127.46	5.39	-1.21	0.69	0.00		
ψ(Elevation),p(Slope)	-127.52	5.60	-1.26	0.67	0.00		
Multi-variable occupancy							
ψ(Bobcat+Trail),p(Slope)	-124.38	5.82	0.00	0.00	0.52	1483.62	0.41
ψ(Slope+NDVI),p(Slope)	-126.25	6.06	-1.88	2.55	0.18	1502.43	0.42
ψ(.),p(Slope)	-126.25	4.08	-1.88	2.52	0.30	1305.13	0.45
ψ(Coyote+Trail),p(Slope)	-126.46	6.24	-2.08	1.60	0.00		
ψ(Bobcat),p(Slope)	-126.74	5.21	-2.37	2.60	0.00		
ψ(Coyote),p(Slope)	-127.25	5.15	-2.87	2.53	0.00		
ψ(Slope+Elevation),p(Slope)	-127.42	6.73	-3.05	2.81	0.00		
ψ(Slope+Canopy),p(Slope)	-127.62	6.36	-3.24	2.62	0.00		
ψ(Bobcat+Coyote),p(Slope)	-127.74	6.46	-3.37	2.63	0.00		
ψ(Bobcat+Canopy),p(Slope)	-128.38	7.01	-4.01	2.75	0.00		
ψ(Canopy+Elevation),p(Slope)	-128.59	6.90	-4.21	2.77	0.00		
ψ(Elevation+NDVI),p(Slope)	-128.67	6.76	-4.29	2.68	0.00		
ψ(Canopy+NDVI),p(Slope)	-128.69	6.70	-4.31	2.61	0.00		

Warm-dry 2018

Model	elpd	nparam	elpd_diff	se_diff	weight	χ2	P value
Detection							
ψ(.),p(Water)	-87.98	3.94	0.00	0.00	0.85		
ψ(.),p(NDVI)	-91.41	4.68	-3.43	2.88	0.13		
ψ(.),p(.)	-91.72	3.54	-3.74	3.16	0.00		
ψ(.),p(Cattle_DR)	-91.99	4.45	-4.01	3.26	0.01		
ψ(.),p(Elevation)	-92.04	5.18	-4.06	3.14	0.00		
ψ(.),p(Bobcat_DR)	-92.40	5.09	-4.42	2.64	0.00		
ψ(.),p(Trail)	-93.32	6.25	-5.34	3.48	0.00		
ψ(.),p(Slope)	-93.99	6.25	-6.01	4.00	0.00		
ψ(.),p(Coyote_DR)	-94.97	7.10	-6.99	4.30	0.00		
Univariate occupancy							
ψ(.),p(Water)	-87.98	3.94	0.00	0.00	0.61		
ψ(Canopy),p(Water)	-88.17	4.77	-0.19	1.05	0.37		
ψ(Water),p(Water)	-88.48	4.94	-0.50	0.81	0.00		
ψ(NDVI),p(Water)	-88.69	5.12	-0.71	1.13	0.02		
ψ(Slope),p(Water)	-88.84	5.30	-0.85	1.25	0.00		
ψ(Elevation),p(Water)	-88.88	5.07	-0.90	0.78	0.00		
ψ(Deciduous),p(Water)	-89.27	5.26	-1.28	0.32	0.00		
ψ(Coniferous),p(Water)	-89.33	5.30	-1.35	0.25	0.00		
Multi-variable occupancy							
ψ(Canopy+NDVI),p(Water)	-87.43	5.67	0.00	0.00	0.51	2347.92	0.32
ψ(Coyote),p(Water)	-87.65	5.14	-0.23	2.33	0.48	2625.30	0.31
ψ(.),p(Water)	-87.98	3.94	-0.55	1.92	0.00		
ψ(Coyote+Canopy),p(Water)	-88.23	6.12	-0.80	2.22	0.00		
ψ(Water+Canopy),p(Water)	-88.55	5.80	-1.12	2.10	0.00		
ψ(Coyote+Trail),p(Water)	-88.62	6.52	-1.19	2.40	0.00		
ψ(Canopy+Slope),p(Water)	-88.79	6.17	-1.37	2.38	0.00		
ψ(Bobcat+Coyote),p(Water)	-88.88	6.38	-1.45	2.49	0.00		
ψ(Bobcat),p(Water)	-89.07	5.10	-1.64	2.05	0.00		
ψ(NDVI+Water),p(Water)	-89.13	6.03	-1.70	2.00	0.00		
ψ(Bobcat+Canopy),p(Water)	-89.15	5.84	-1.72	1.87	0.00		
ψ(Bobcat+Trail),p(Water)	-89.19	6.18	-1.76	2.25	0.00		

Rainy 2018

Model	elpd	nparam	elpd_diff	se_diff	weight	χ2	P value
Detection							
ψ(.),p(Water)	-103.88	4.08	0.00	0.00	0.62		
ψ(.),p(Slope)	-104.24	4.48	-0.35	1.49	0.15		
ψ(.),p(.)	-104.27	3.32	-0.38	1.53	0.00		
ψ(.),p(Cattle_DR)	-104.91	4.66	-1.03	2.21	0.22		
ψ(.),p(NDVI)	-105.47	5.30	-1.58	1.82	0.00		
ψ(.),p(Elevation)	-105.89	5.21	-2.01	1.79	0.00		
ψ(.),p(Trail)	-106.19	5.55	-2.30	2.64	0.00		
ψ(.),p(Coyote_DR)	-106.80	7.32	-2.92	2.56	0.00		
ψ(.),p(Bobcat_DR)	-108.65	8.86	-4.77	2.78	0.00		
Univariate occupancy							
ψ(.),p(Water)	-103.88	4.08	0.00	0.00	0.51		
ψ(Elevation),p(Water)	-104.06	5.15	-0.18	1.43	0.42		
ψ(NDVI),p(Water)	-104.42	5.23	-0.54	1.24	0.07		
ψ(Water),p(Water)	-104.66	5.14	-0.78	0.79	0.00		
ψ(Coniferous),p(Water)	-104.97	5.20	-1.09	0.29	0.00		
ψ(Slope),p(Water)	-105.07	5.29	-1.19	0.24	0.00		
ψ(Deciduous),p(Water)	-105.14	5.38	-1.26	0.32	0.00		
ψ(Canopy),p(Water)	-105.54	5.90	-1.66	0.75	0.00		
Multi-variable occupancy							
ψ(Coyote),p(Water)	-103.83	5.11	0.00	0.00	0.49	4081.87	0.12
ψ(.),p(Water)	-103.88	4.08	-0.05	1.63	0.46	4088.34	0.13
ψ(NDVI),p(Water)	-104.37	5.16	-0.54	1.66	0.00		
ψ(Bobcat+Coyote),p(Water)	-104.49	6.18	-0.66	1.33	0.05	4280.14	0.12
ψ(Bobcat),p(Water)	-104.86	5.38	-1.03	1.84	0.00		
ψ(Slope),p(Water)	-105.04	5.24	-1.21	1.64	0.00		
ψ(Coyote+Trail),p(Water)	-105.23	6.68	-1.40	0.73	0.00		
ψ(Coyote+Canopy),p(Water)	-105.31	6.75	-1.48	0.85	0.00		
ψ(Canopy),p(Water)	-105.59	5.94	-1.76	1.83	0.00		
ψ(Bobcat+Trail),p(Water)	-105.69	6.64	-1.86	1.76	0.00		
ψ(Canopy+NDVI),p(Water)	-106.14	7.22	-2.31	1.80	0.00		
ψ(Bobcat+Canopy),p(Water)	-106.44	7.09	-2.61	2.00	0.00		
ψ(Canopy+Slope),p(Water)	-106.77	7.15	-2.94	1.90	0.00		

Cold-dry 2018

Model	elpd	nparam	elpd_diff	se_diff	weight	χ2	P value
Detection							
ψ(.),p(Elevation)	-111.24	7.51	0.00	0.00	0.49		
ψ(.),p(Water)	-115.66	5.49	-4.42	9.67	0.51		
ψ(.),p(Bobcat_DR)	-116.95	6.23	-5.71	6.82	0.00		
ψ(.),p(.)	-116.96	3.86	-5.71	9.51	0.00		
ψ(.),p(NDVI)	-118.01	5.12	-6.77	9.26	0.00		
ψ(.),p(Cattle_DR)	-118.37	5.97	-7.13	9.33	0.00		
ψ(.),p(Coyote_DR)	-118.37	5.27	-7.13	9.50	0.00		
ψ(.),p(Slope)	-119.64	7.61	-8.40	9.81	0.00		
ψ(.),p(Trail)	-119.81	7.59	-8.57	10.21	0.00		
Univariate occupancy							
ψ(Canopy),p(Elevation)	-111.18	8.53	0.00	0.00	0.48		
ψ(.),p(Elevation)	-111.24	7.51	-0.06	1.97	0.00		
ψ(Slope),p(Elevation)	-111.27	8.34	-0.09	2.35	0.12		
ψ(Elevation),p(Elevation)	-111.34	8.42	-0.16	2.81	0.37		
ψ(Deciduous),p(Elevation)	-111.35	8.48	-0.17	2.56	0.03		
ψ(Coniferous),p(Elevation)	-111.74	8.36	-0.56	2.30	0.00		
ψ(NDVI),p(Elevation)	-111.77	7.96	-0.59	1.92	0.00		
ψ(Water),p(Elevation)	-112.10	8.33	-0.92	2.01	0.00		
Multi-variable occupancy							
ψ(Canopy+Elevation),p(Elevation)	-110.96	9.76	0.00	0.00	0.55	1238.475	0.41
ψ(Canopy),p(Elevation)	-111.18	8.53	-0.22	1.91	0.00		
ψ(Canopy+Coyote),p(Elevation)	-111.32	9.33	-0.36	2.67	0.44	994.56	0.45
ψ(Canopy+NDVI),p(Elevation)	-111.67	9.04	-0.71	1.90	0.00		
ψ(Canopy+Elevation+Slope),p(Elevation)	-111.83	10.96	-0.87	1.05	0.01	1220.20	0.43
ψ(Canopy+Elevation+NDVI),p(Elevation)	-112.12	10.94	-1.16	0.50	0.00		
ψ(Canopy+Cattle),p(Elevation)	-112.26	9.59	-1.30	2.07	0.00		
ψ(Canopy+Coyote+Trail),p(Elevation)	-112.47	10.55	-1.51	2.65	0.00		
ψ(Canopy+Slope),p(Elevation)	-112.69	10.50	-1.73	2.04	0.00		
ψ(Canopy+NDVI+Slope),p(Elevation)	-112.75	10.76	-1.79	2.20	0.00		
ψ(Canopy+Bobcat+Coyote),p(Elevation)	-112.77	10.73	-1.81	2.81	0.00		
ψ(Canopy+Bobcat),p(Elevation)	-112.92	10.34	-1.96	1.82	0.00		
ψ(Canopy+Bobcat+Trail),p(Elevation)	-113.04	10.56	-2.08	2.30	0.00		

Ground Squirrel

Warm-dry 2018

Model	elpd	nparam	elpd_diff	se_diff	weight	χ2	*P* value
Detection							
ψ(.),p(Water)	-78.95	3.73	0.00	0.00	0.44		
ψ(.),p(VRM)	-79.25	3.99	-0.31	2.62	0.43		
ψ(.),p(.)	-79.47	2.78	-0.52	1.84	0.00		
ψ(.),p(Coyote_DR)	-79.49	3.31	-0.54	1.86	0.00		
ψ(.),p(Elevation)	-79.68	3.57	-0.73	2.10	0.00		
ψ(.),p(Bobcat_DR)	-80.36	4.56	-1.41	3.08	0.13		
ψ(.),p(NDVI)	-80.53	3.76	-1.58	1.68	0.00		
ψ(.),p(Cattle_DR)	-80.71	3.93	-1.77	2.08	0.00		
ψ(.),p(Trail)	-81.24	4.89	-2.29	2.44	0.00		
Univariate occupancy							
ψ(.),p(Water)	-78.95	3.73	0.00	0.00	0.80		
ψ(Canopy),p(Water)	-79.29	4.70	-0.35	0.97	0.20		
ψ(Elevation),p(Water)	-79.88	4.85	-0.93	0.77	0.00		
ψ(Coniferous),p(Water)	-80.01	5.06	-1.06	0.74	0.00		
ψ(Water),p(Water)	-80.02	4.99	-1.07	0.43	0.00		
ψ(NDVI),p(Water)	-80.19	5.06	-1.24	0.48	0.00		
ψ(VRM),p(Water)	-80.21	5.01	-1.26	0.39	0.00		
ψ(Deciduous),p(Water)	-80.33	5.18	-1.38	0.35	0.00		
Multi-variable occupancy							
ψ(.),p(Water)	-78.95	3.73	0.00	0.00	0.52	2399.51	0.30
ψ(Bobcat),p(Water)	-79.00	4.83	-0.05	1.36	0.30	2348.14	0.28
ψ(Bobcat+Coyote),p(Water)	-79.74	6.15	-0.80	1.78	0.05	2633.09	0.24
ψ(Bobcat+Trail),p(Water)	-79.88	6.31	-0.94	1.75	0.03	2633.09	0.24
ψ(Coyote),p(Water)	-79.96	5.07	-1.01	0.74	0.00		
ψ(Cattle),p(Water)	-80.17	4.93	-1.23	0.44	0.00		
ψ(Canopy+NDVI),p(Water)	-80.18	6.08	-1.24	1.37	0.10		
ψ(Bobcat+VRM),p(Water)	-80.23	6.06	-1.29	1.44	0.00		
ψ(Canopy+Water),p(Water)	-80.55	6.29	-1.60	1.23	0.00		
ψ(Coyote+Canopy),p(Water)	-80.60	6.19	-1.65	1.22	0.00		
ψ(Coyote+Trail),p(Water)	-81.01	6.89	-2.06	1.77	0.00		
ψ(NDVI+Water),p(Water)	-81.39	6.47	-2.45	0.76	0.00		
ψ(Cattle+NDVI),p(Water)	-81.50	6.36	-2.55	0.72	0.00		
ψ(VRM+Water),p(Water)	-81.50	6.57	-2.55	0.67	0.00		

Rainy 2018

Model	elpd	nparam	elpd_diff	se_diff	weight	χ2	*P* value
Detection							
ψ(.),p(Bobcat_DR)	-98.75	7.89	0.00	0.00	0.61		
ψ(.),p(Cattle_DR)	-100.56	7.52	-1.81	7.00	0.03		
ψ(.),p(VRM)	-100.67	5.52	-1.92	8.04	0.21		
ψ(.),p(NDVI)	-104.29	7.34	-5.54	9.32	0.15		
ψ(.),p(.)	-104.89	4.34	-6.14	9.47	0.00		
ψ(.),p(Water)	-106.16	6.85	-7.41	9.02	0.00		
ψ(.),p(Coyote_DR)	-106.36	6.89	-7.61	9.10	0.00		
ψ(.),p(Trail)	-107.20	7.94	-8.45	10.94	0.00		
ψ(.),p(Elevation)	-110.24	11.00	-11.49	10.92	0.00		
Univariate occupancy							
ψ(Water),p(Bobcat_DR)	-98.00	9.24	0.00	0.00	0.65		
ψ(NDVI),p(Bobcat_DR)	-99.67	9.34	-1.67	2.12	0.00		
ψ(Elevation),p(Bobcat_DR)	-99.95	9.49	-1.95	2.68	0.01		
ψ(Coniferous),p(Bobcat_DR)	-100.03	9.26	-2.03	2.35	0.00		
ψ(Canopy),p(Bobcat_DR)	-100.29	9.53	-2.28	2.18	0.00		
ψ(Deciduous),p(Bobcat_DR)	-100.43	9.60	-2.43	2.18	0.00		
ψ(VRM),p(Bobcat_DR)	-100.47	10.08	-2.46	2.07	0.00		
ψ(.),p(Bobcat_DR)	-100.67	5.52	-2.66	8.77	0.34		
Multi-variable occupancy							
ψ(Water+Coyote+Trail),p(Bobcat_DR)	-97.32	10.62	0.00	0.00	0.28	1056.21	0.73
ψ(Water+Cattle),p(Bobcat_DR)	-97.75	10.21	-0.42	1.31	0.21	1119.00	0.72
ψ(Water+Coyote),p(Bobcat_DR)	-98.00	10.45	-0.67	1.00	0.00		
ψ(Water),p(Bobcat_DR)	-98.00	9.24	-0.68	1.70	0.33	1034.75	0.73
ψ(Water+Bobcat+Coyote),p(Bobcat_DR)	-98.32	11.25	-1.00	1.69	0.17	990.64	0.74
ψ(Water+Canopy),p(Bobcat_DR)	-98.81	10.30	-1.49	1.80	0.00		
ψ(Water+Coyote+Canopy),p(Bobcat_DR)	-98.94	11.35	-1.61	1.11	0.00		
ψ(Water+VRM),p(Bobcat_DR)	-99.06	10.52	-1.73	1.81	0.00		
ψ(Water+NDVI),p(Bobcat_DR)	-99.25	10.81	-1.92	1.64	0.00		
ψ(Water+Bobcat),p(Bobcat_DR)	-99.46	10.99	-2.13	1.94	0.00		
ψ(Water+Cattle+NDVI),p(Bobcat_DR)	-99.77	12.39	-2.45	2.23	0.00		
ψ(Water+Bobcat+VRM),p(Bobcat_DR)	-99.87	11.49	-2.55	2.03	0.00		
ψ(Water+Bobcat+Trail),p(Bobcat_DR)	-99.91	11.73	-2.59	2.17	0.00		
ψ(Water+Canopy+NDVI),p(Bobcat_DR)	-100.47	12.47	-3.15	1.52	0.00		
ψ(Water+NDVI+VRM),p(Bobcat_DR)	-101.05	12.79	-3.72	1.64	0.00		
